# Genetic scores for adult subcortical volumes associate with subcortical volumes during infancy and childhood

**DOI:** 10.1002/hbm.25292

**Published:** 2021-02-02

**Authors:** Sander Lamballais, Philip R. Jansen, Jeremy A. Labrecque, M. Arfan Ikram, Tonya White

**Affiliations:** ^1^ Department of Epidemiology Erasmus MC University Medical Center Rotterdam Rotterdam the Netherlands; ^2^ Department of Complex Trait Genetics, Center for Neurogenomics and Cognitive Research, Amsterdam Neuroscience VU University Amsterdam the Netherlands; ^3^ Department of Clinical Genetics, VU Medical Center Amsterdam UMC Amsterdam the Netherlands; ^4^ Department of Child and Adolescent Psychiatry Erasmus MC University Medical Center Rotterdam Rotterdam the Netherlands; ^5^ Department of Radiology and Nuclear Medicine Erasmus MC University Medical Center Rotterdam Rotterdam the Netherlands

**Keywords:** childhood, infancy, MRI, polygenic scores, subcortical volume, ultrasound

## Abstract

Individual differences in subcortical brain volumes are highly heritable. Previous studies have identified genetic variants that underlie variation in subcortical volumes in adults. We tested whether those previously identified variants also affect subcortical regions during infancy and early childhood. The study was performed within the Generation R study, a prospective birth cohort. We calculated polygenic scores based on reported GWAS for volumes of the accumbens, amygdala, brainstem, caudate nucleus, globus pallidus, putamen, and thalamus. Participants underwent cranial ultrasound around 7 weeks of age (range: 3–20), and we obtained metrics for the gangliothalamic ovoid, a predecessor of the basal ganglia. Furthermore, the children participated in a magnetic resonance imaging (MRI) study around the age of 10 years (range: 9–12). A total of 340 children had complete data at both examinations. Polygenic scores primarily associated with their corresponding volumes at 10 years of age. The scores also moderately related to the diameter of the gangliothalamic ovoid on cranial ultrasound. Mediation analysis showed that the genetic influence on subcortical volumes at 10 years was only mediated for 16.5–17.6% of the total effect through the gangliothalamic ovoid diameter at 7 weeks of age. Combined, these findings suggest that previously identified genetic variants in adults are relevant for subcortical volumes during early life, and that they affect both prenatal and postnatal development of the subcortical regions.

## Graphical abstract

We show that polygenic scores for adult subcortical volumes relate to subcortical volume during infancy and childhood. Furthermore, the polygenic scores seemed to associate partly independent with infant and childhood subcortical volumes, suggesting that the polygenic scores capture specific stages of postnatal development.

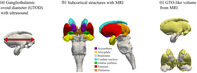

## 1 INTRODUCTION

Subcortical brain structures are a collection of diverse regions with a myriad of functions. Subtle differences in these regions have been implicated in cognitive function, emotion regulation, and certain psychiatric disorders like schizophrenia and autism spectrum disorder (Janacsek et al., 2020; Tian et al., 2017; van Erp et al., 2016; van Rooij et al., 2018). Similar to the rest of the brain (Gennatas et al., 2017; Somerville, 2016; Stiles & Jernigan, 2010; Walhovd et al., 2011), the subcortex develops over a lifetime, with emphasis on expansive growth during the prenatal period, reorganization during early life and atrophy during late life. The development of subcortical regions is partly shaped by stochastic events (White, 2019) and by environmental factors such as early‐life adverse events (Calem, Bromis, McGuire, Morgan, & Kempton, 2017; Cassiers et al., 2018; Frodl et al., 2017). Subcortical development is also strongly influenced by genetics, as the heritability has been estimated to be around 60–80% for subcortical volumes (Batouli, Trollor, Wen, & Sachdev, 2014; A. G. Jansen, Mous, White, Posthuma, & Polderman, 2015). Further elucidating the role of genetics in the developmental trajectories of subcortical regions may in turn provide insight into the functional consequences of the subcortex.

The strong heritability of subcortical volumes likely reflects the combined effect of weak signals across the genome rather than a single gene. This was confirmed through genome‐wide association studies (GWAS) (Hibar et al., 2015), which aimed to establish associations between a given phenotype and a wide range of markers of genomic variation such as single nucleotide polymorphisms (SNPs). In 2015, Hibar and colleagues performed GWAS on the volumes of several subcortical regions, and identified a total of seven SNPs that specifically related to the volumes of either the putamen, the caudate nucleus or the hippocampus (Hibar et al., 2015). More recently, Satizabal et al. (2019) performed a GWAS in over 40,000 adults and extended the findings to 25 genetic loci, potentially implicating 62 different genes. These loci provide an opportunity to further understand the genetic influences underlying subcortical development.

The GWAS informs about the genetic underpinnings of subcortical volumes, but its study population consisted primarily of adults. It is not clear whether these findings would generalize for studies on subcortical volumes during childhood or even infancy. The GWAS likely captured signal that related to all aspects of changes in subcortical volumes, that is, early‐life development, the height of the peak in subcortical volume and the degenerative processes leading to a decline in volume with age. Furthermore, children may reach the same peak subcortical volume during adulthood, but may do so at different rates, which would also further diminish the value of the identified genetic markers in a pediatric population.

We therefore aimed to study two related questions. First, we aimed to study whether the genetic markers that were identified during adulthood relate to subcortical volumes earlier in life, that is, infancy and early childhood. Second, assuming that the genetic markers do associate with early‐life subcortical volumes, it remains unclear during which life phase the genetic effects primarily take place. Subcortical volumes during infancy likely correlate strongly to subcortical volumes during early childhood. If the genetic effects mainly occur during fetal development, then the associations would still carry over to early childhood. We would want to study whether the genetic markers still associate with subcortical volumes during early childhood when considering subcortical volumes during infancy.

Within this context, we aimed to study during which life phases the adult‐derived genomic loci are relevant. We performed the current study in the Generation R Study, a prospective birth cohort based in Rotterdam, the Netherlands (Tiemeier et al., 2012). A subset of the children of Generation R were invited for a cranial ultrasound around 7 weeks of age. From these images we quantified the gangliothalamic ovoid (GTO) diameter (GTOD), the diameter of a subcortical structure that develops into the basal ganglia. At around 10 years of age, the children were additionally invited for an MRI scan of the brain. Next, we utilized a technique known as polygenic scores (PGS), a score calculated based on GWAS results that quantifies a person's genetic predisposition for a trait, in this case the subcortical volumes. Within Generation R we obtained genotypic information, and for each child we calculated PGS for each subcortical region. We then assessed whether the PGS associated with the ultrasound metrics during infancy and the MRI scan during childhood. Finally, to see whether the PGS associations with subcortical volumes during childhood were mediated by the PGS associations with infant subcortical volumes we formulated a causal mediation model. Importantly, the GTOD measurement is coarser than the MRI measurements given that the GTOD is a diameter while the MRI measurements are volumetric, like the original GWAS. While this will reduce the accuracy to estimate the mediation, we do not expect systematic noise in the GTOD measurement that could bias the estimation. Furthermore, the analysis should provide some insight into whether the infant subcortical measures mediate the association between the PGS and the childhood subcortical volumes.

## 2 METHODS

### 2.1 Study population

**FIGURE 1:**
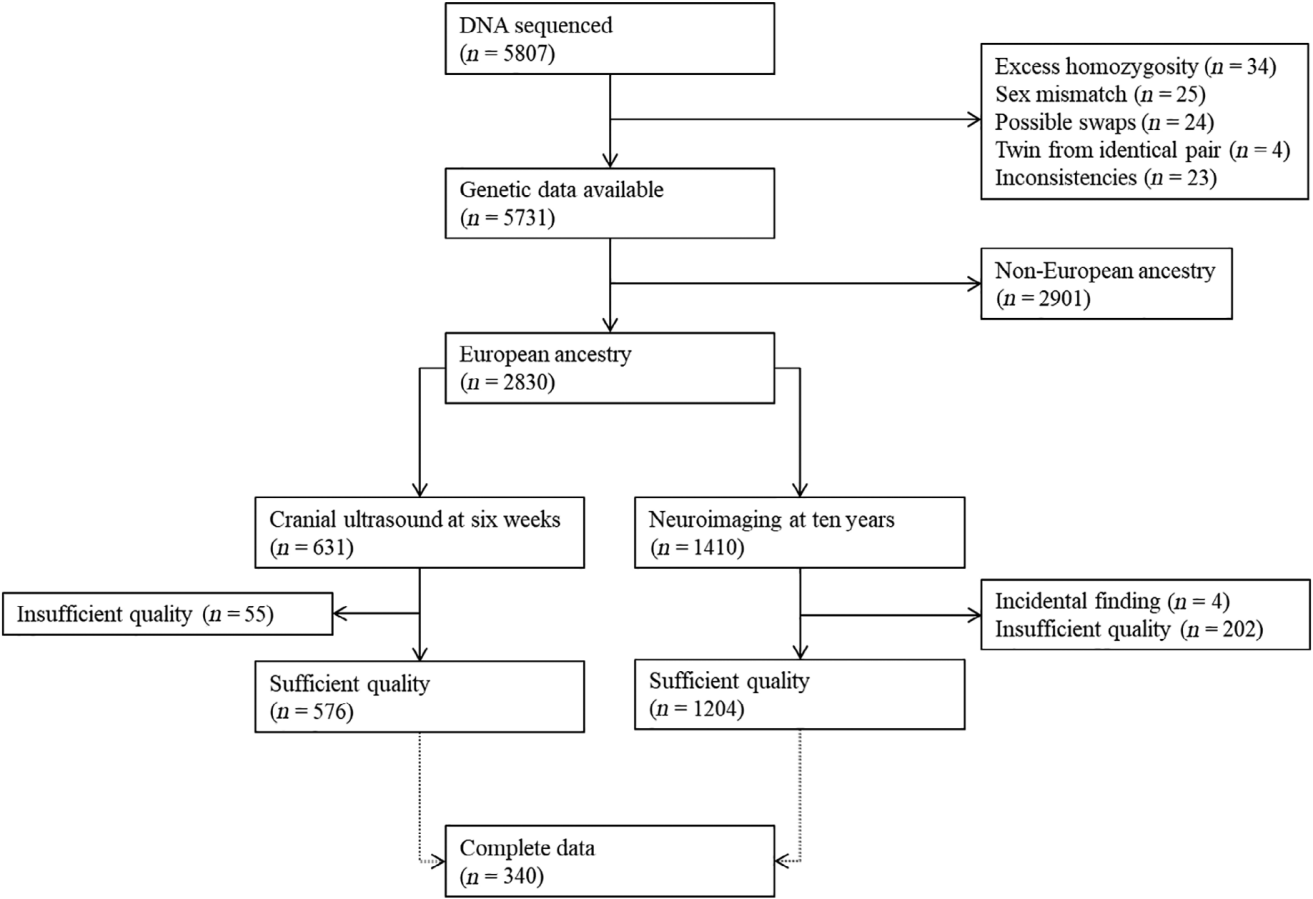
Flowchart of the study population

The study was conducted within the Generation R cohort, a prospective birth cohort based in Rotterdam, the Netherlands (Tiemeier et al., 2012). At approximately 7 weeks of age, a subset of the children partook in cranial ultrasound measurements. At approximately 10 years of age, most of these children also visited the research center for MRI scans of the brain (White et al., 2017). A flowchart of the sample is shown in Figure 1. Out of 2,830 children of European ancestry and with genetic data available, 576 had data on the 7‐week ultrasound and 1,204 had data on the 10‐year MRI. In total, 340 children had data on both measures.

### 2.2 Ethics statement

The study was approved by the Medical Ethical Committee of the Erasmus MC University Medical Center in Rotterdam, and written informed consent was obtained from all primary caregivers of the participants.

### 2.3 Genotyping and polygenic risk scores

All genotyping and quality control procedures have been described elsewhere (Jaddoe et al., 2007; Medina‐Gomez et al., 2015). Samples were collected from cord blood at birth (Illumina 610K Quad Chip) or from venipuncture at a visit to the research center at the age of around 6 years (Illumina 660K Quad Chip). The Illumina 610K and 660K samples were merged based on their overlapping SNPs. Quality control was performed using PLINK (version 1.9; Purcell et al., 2007). SNPs were removed if the minor allele frequency was below 1%, the Hardy–Weinberg disequilibrium *p*‐value below >1 × 10^−6^ or a SNP call rate of less than 98%. Individual data were removed in cases of genetic and phenotypic sex mismatch, excess rates of homozygosity of the genotypes (>4 *SD*) and genotype quality (>5% missing). Pairs of individuals with identical genetic information but who were not identical twins were removed, as they were likely samples that were processed twice. For identical twins, the twin with the lower call rate was removed.

The current study was based on a GWAS on subcortical volumes (Satizabal et al., 2019). The GWAS included 40,000 participants of predominantly European ancestry, and they did not overlap with the present study sample. They performed a GWAS for each subcortical volume. We utilized the summary statistics to calculate a PGS per subcortical region. PGS can be calculated by summing the effect size of each SNP for a given person (Vilhjalmsson et al., 2015). However, SNPs have proximal dependence and tend to co‐occur the closer they are to each other, a principle known as linkage disequilibrium (LD). A common method to adjust for this is a sliding window approach, where the SNP with the lowest p‐value within that range is kept and all other SNPs are pruned. We used a more refined method named LDpred (Vilhjalmsson et al., 2015), which utilizes LD information from an external dataset to improve prediction accuracy. Furthermore, different scores can be calculated depending on the fraction *P* SNPs that are assumed to be causal. We used different values for the *P* parameter: 1.0, 0.5, 0.1, 0.05, 0.01, and 0.005. The 1000 Genomes project was used as the external LD dataset (The 1000 Genomes Project Consortium, 2015). To account for the European ancestry in the original GWAS we calculated the genomic components of our study population with the multi‐dimensional scaling function of PLINK (Medina‐Gomez et al., 2015; Purcell et al., 2007), and we subsequently selected participants of European ancestry.

### 2.4 Ultrasound image acquisition and processing

**FIGURE 2:**
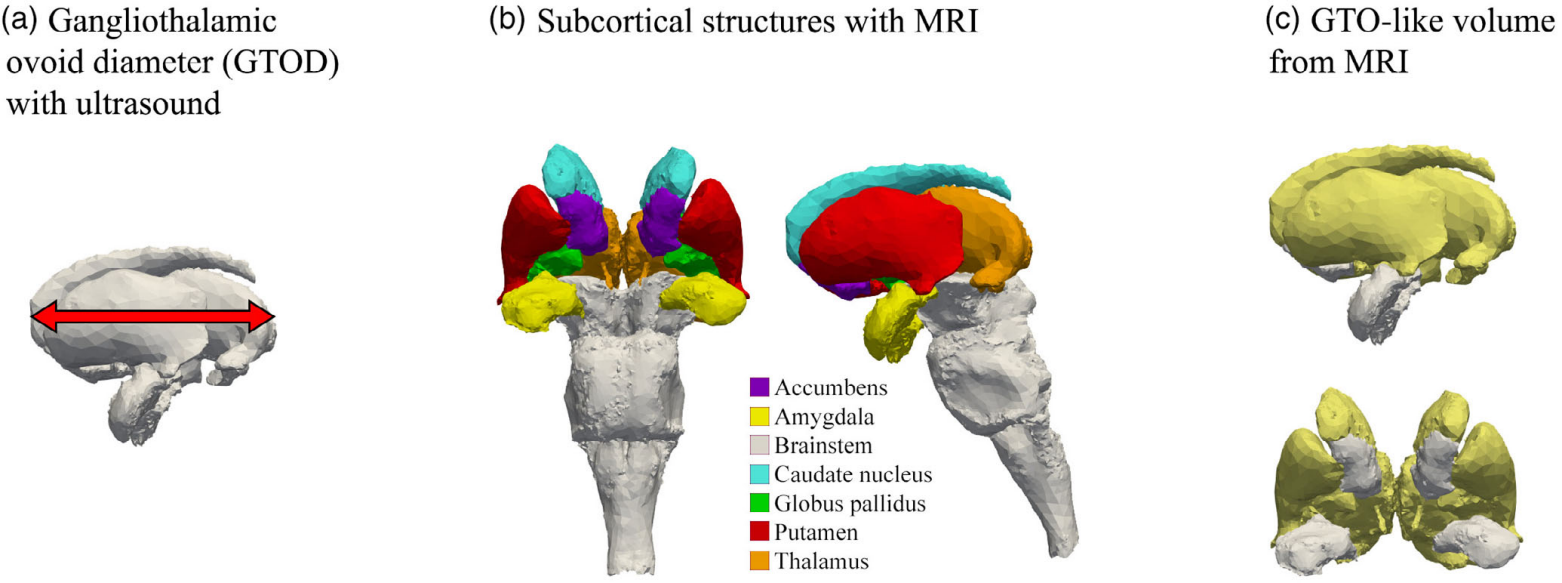
Visual representation of the subcortical structures under study. Panel a shows how the gangliothalamic ovoid diameter was determined from the ultrasound at 7 weeks. Panel b displays the subcortical regions that were obtained from the MR images at the 10 year visit. Panel c shows how we approximated a GTO‐like volume using the MR image data, that is, by summing the volumes for the caudate nucleus, the globus pallidus, the putamen and the thalamus. The images were based on data from the MIDA model (Iacono et al., 2015)

Three‐dimensional cranial ultrasound was performed at approximately 7 weeks of postnatal age using a multifrequency electronic transducer (3.7–9.3 MHz) with a scan angle of 146° (Voluson 730 Expert, GE Healthcare, Waukesha, WI). The procedure has been described elsewhere (Herba et al., 2010; Roza et al., 2008). In brief, the probe was placed on the anterior fontanel. The volume box was positioned at the level of the foramen of Monro, and a pyramid‐shaped volume of brain tissue was imaged. Images were subsequently used for measuring the GTOD (Figure 2a), which was done by two trained raters. The reliability of the measurements was high (Cronbach's *α* = .83), and thus the average of the diameters of the two raters were used for analysis.

### 2.5 MRI image acquisition and processing

The MRI imaging and image processing at the 10‐year visit has been described elsewhere (White et al., 2017). In brief, structural brain images were obtained on a 3T GE Discovery MR750w MRI System (General Electric, Milwaukee, WI) using an 8‐channel receive‐only head coil. T_1_‐weighted images were obtained using an inversion recovery‐prepared fast spoiled gradient recalled (IF‐SPGR) sequence (T_R_ = 8.77 ms, T_E_ = 3.4 ms, T_I_ = 600 ms, flip angle = 10°, field of view = 220 × 220 mm, acquisition matrix = 220 × 220, slice thickness = 1 mm, number of slices = 230, bandwidth = 25 kHz). The images were processed through the Freesurfer analysis suite, version 6.0 (Fischl et al., 2004). After removal of nonbrain tissue and normalizing voxel intensities for B_1_ homogeneities, the images were segmented into prespecified cortical and subcortical regions and their volumes were determined. We extracted summary information on volume of the nucleus accumbens, the amygdala, the brainstem, the caudate nucleus, the globus pallidus, the putamen and the thalamus (Figure 2b). We averaged the volume of the left and right hemispheric subcortical structures as this was done in the original GWAS on subcortical volume as well and we did not expect a lateralized effect.

### 2.6 Statistical analysis

The study focused on four sets of analyses. Analyses were performed using R, version 3.5.1 (R Core Team, 2016).

#### 2.6.1 Subcortical PGS and childhood MRI

In the first set of analyses, we used linear regression models to explore whether PGS for subcortical regions associated with subcortical volumes obtained with MRI at around 10 years of age. We first assessed whether PGS and volumes for the same region were associated (within‐region associations). Next, we considered whether PGS and volumes of differing regions were associated (between‐region associations). We created a separate linear regression for each combination of PGS and subcortical volumes across all values for parameter *P*, yielding 7 × 7 × 6 = 294 models. In each model, the PGS and the volumes were standardized in order to increase comparability of the findings. Furthermore, to control for potential confounding, we adjusted for age during the MRI (in years), intracranial volume (in ml), sex and the first 10 genomic components. To adjust for multiple testing, we applied a Bonferroni correction to the 7 × 7 different models within a given parameter *P*. The PGS based on different values for parameter *P* tended to correlate very strongly (median Pearson's *r* = .91), thus due to the conservative nature of the Bonferroni correction, we did not additionally correct for each parameter of *P*. The alpha level was thus set at .05/49 = .00102.

#### 2.6.2 GTO PGS and infant US

For the second set of analyses, we used linear regression models to see whether the subcortical PGS associated with GTOD as obtained from the ultrasound during infancy. As the GTO is a combination of different subcortical regions, we created a PGS for the GTO by summing the PGS of several regions together. Given that these subcortical regions differ in size, we calculated a relative GTO PGS according to the following formula:

$${\mathrm{PGS}}_{\left(\mathrm{GTO}\right)}=0.22\cdot {\mathrm{PGS}}_{\left(\text{caudate}\right)}+0.10\cdot {\mathrm{PGS}}_{\left(\text{pallidus}\right)}+0.28\cdot {\mathrm{PGS}}_{\left(\text{putamen}\right)}+0.40\cdot {\mathrm{PGS}}_{\left(\text{thalamus}\right)}$$

The weights were obtained by considering the mean relative size of the subcortical regions as obtained from the MRI around the age of 10 years, as we did not have information on the relative size of these regions from the ultrasound during infancy. Similar to the first set of analyses, the variables were standardized, and the models were corrected for gestational age at birth (in weeks), age at ultrasound (in weeks), head circumference (in cm), sex, and the first 10 genomic components. Given that only one analysis was performed per value for parameter *P*, we did not apply correction for multiple testing and the alpha level was set at .05.

#### 2.6.3 GTO PGS and childhood MRI

As a third set of analyses, we wanted to assess whether the GTO PGS associated with the equivalent of the GTO at age 10 years. We therefore calculated a GTO‐like volume, where the volumes of the caudate nucleus, the pallidi, the putamen and the thalami were summed together (Figure 2c). Then, we used linear regression to assess the association between the GTO PGS and this GTO‐like volume. The models were specified similarly as the first set of analyses. Given that only one analysis was performed per value for parameter *P*, we did not apply correction for multiple testing and the alpha level was set at .05.

#### 2.6.4 Mediation analyses

For the fourth set of analyses, we explored whether the associations between the PGS and the subcortical volumes at 10 years of age were mediated by the GTOD during infancy. The genetic loci captured by the PGS may drive both prenatal and postnatal effects on neurodevelopment. Any relevance to prenatal development should be expressed by stronger associations between a GTO PGS and the GTOD from the postnatal ultrasound at 7 weeks than the association between a GTO PGS and the GTO‐like volume from the MRI at 10 years. More critically, if the PGS primarily affects postnatal development then the PGS should associate with GTO‐like volume from MRI at 10 years when also considering the GTOD from the postnatal ultrasound at 7 weeks. We therefore created a causal mediation model with the GTO PGS as the determinant, the GTOD from the 7‐weeks postnatal ultrasound as the mediator and the GTO‐like volume at age 10 as the outcome (VanderWeele, 2016). Causal mediation allows for estimation of the relative contributions of the natural direct effect of determinant on the outcome and the natural indirect effect that runs from the determinant to the outcome through the mediator. The confidence intervals were estimated through nonparametric bootstrapping. Causal mediation assumes that all confounding factors for the direct effect and the indirect effect are accounted for. We therefore corrected for sex, age, intracranial volume, and the first ten genomic components when estimating the exposure‐outcome association. For the mediator‐outcome association we additionally corrected for maternal education at birth of the child (low, medium, or high) and birth weight of the child in grams. The analyses were performed using the R “mediation” package (Tingley, Yamamoto, Hirose, Keele, & Imai, 2014).

#### 2.6.5 Samples used in the analyses

The main measurements—that is, cord blood at birth or venipuncture at the research center, postnatal ultrasound and the MRI visit—were performed at very different phases of the children's lives. In addition, the postnatal ultrasound was only administered in a subsample of the whole Generation R study. As such, the number of children with complete data was relatively small compared to the number of children that had genetic data and one of the two imaging measures (Figure 1). The sample size therefore differs per analysis, with 1,204 children for the PGS‐MRI associations, 576 children for the PGS‐GTOD associations, and 340 children for the PGS‐MRI associations mediated by the GTOD.

#### 2.6.6 Sensitivity analyses

Previous literature suggests that subcortical development differs between men and women, with local differences in rate and variability independent from total brain size (Herting et al., 2018; Kiraly et al., 2016; Raznahan et al., 2014; Wierenga et al., 2018). We reanalyzed the PGS and MRI subcortical volume associations by adding an interaction term for the different PGS and sex. Furthermore, we reanalyzed all associations using only the set of 340 children with complete data. Finally, to make the GTO‐like volume more comparable to the GTOD in the mediation analyses, we modeled the GTO‐like volume to as a spherical volume to derive a diameter. The estimated diameter was defined as $2\sqrt[{3}]{\frac{3v}{4\pi }}$, where *v* was the GTO‐like volume.

To assure that the FreeSurfer subcortical segmentations were of good quality, each subcortical structure segmentation was visually inspected in 929 of the 1201 children (Weeland et al., 2020). Most regions had >98% children with sufficient quality, except for the caudate (94.4%), the putamen (88.3%), and the pallidum (90.9%). We therefore performed stringent sensitivity analyses where children were only included if their subcortical segmentation had been assessed, and if they passed quality assessment for all regions, that is, 761 out of 929 children. In the 340 children with both ultrasound and MRI data, 272 had their segmentation quality rated, and of those 219 had sufficient segmentation quality to be included in the sensitivity analysis.

## 3 RESULTS

### 3.1 Characteristics of the study population

The population characteristics are displayed in Table 1. In the whole study population, the cranial ultrasound took place at a mean age of 6.8 (*SD* = 1.8) weeks, with a mean head circumference of 38.6 (*SD* = 1.5) cm and a mean GTOD of 4.3 (*SD* = 0.2) cm. Participants on average were 10.2 (*SD* = 0.6) years of age during the MRI scan, with a mean intracranial volume of 1,540 (*SD* = 138) ml. PGS at different parameter *P* values for any given region were highly correlated (median Pearson's *r* = 0.91; Figure S1), also in those with only complete data (Figure S2).

**TABLE 1:** Characteristics of the study population

	Partial data set (*n* = 1,430)	Complete data set (*n* = 340)
General characteristics		
Gender, boy (*n*, %)	730 (51.0%)	169 (49.7%)
Gestational age at birth (weeks)	40.1 (1.6)	40.2 (1.5)
Birth weight (g)^a^	3,533 (524)	3,530 (510)
Maternal age at birth (years)^a^	32.1 (4.0)	32.2 (3.9)
Maternal education at birth (*n*, %)^a^		
Low	118 (8.4%)	29 (8.6%)
Middle	321 (22.8%)	69 (20.5%)
High	966 (68.8%)	238 (70.8%)
Ultrasound (*n*)	566	340
Age (weeks)	6.8 (1.8)	6.5 (1.6)
Head circumference (cm)	38.6 (1.5)	38.5 (1.5)
GTO diameter (cm)	4.3 (0.2)	4.3 (0.2)
Magnetic resonance imaging (*n*)	1,201	340
Age (years)	10.2 (0.6)	10.2 (0.6)
Intracranial volume (ml)	1,540 (138)	1,531 (138)
Accumbens nucleus volume (ml)	1.4 (0.2)	1.4 (0.2)
Amygdala volume (ml)	3.6 (0.4)	3.6 (0.4)
Brainstem volume (ml)	18.9 (1.8)	18.9 (1.7)
Caudate nucleus volume (ml)	8.3 (1.0)	8.2 (0.9)
Globus pallidus volume (ml)	3.9 (0.4)	3.9 (0.4)
Putamen volume (ml)	10.8 (1.1)	10.8 (1.1)
Thalamus volume (ml)	15.2 (1.3)	15.1 (1.3)

*Note:* The values represent the means and *SD*s unless stated otherwise.

^a^ These variables are not included in the analyses, but are used to show the differences between the partial and complete sets.

Participants with complete data did not differ from participants with data on only MRI characteristics. However, compared to participants with only ultrasound they were slightly younger (mean difference = 0.3 weeks, *p* < .001) and had a smaller head circumference (mean difference = 0.1 cm, *p* = .016).

### 3.2 Subcortical volume PGS and childhood MRI

**FIGURE 3:**
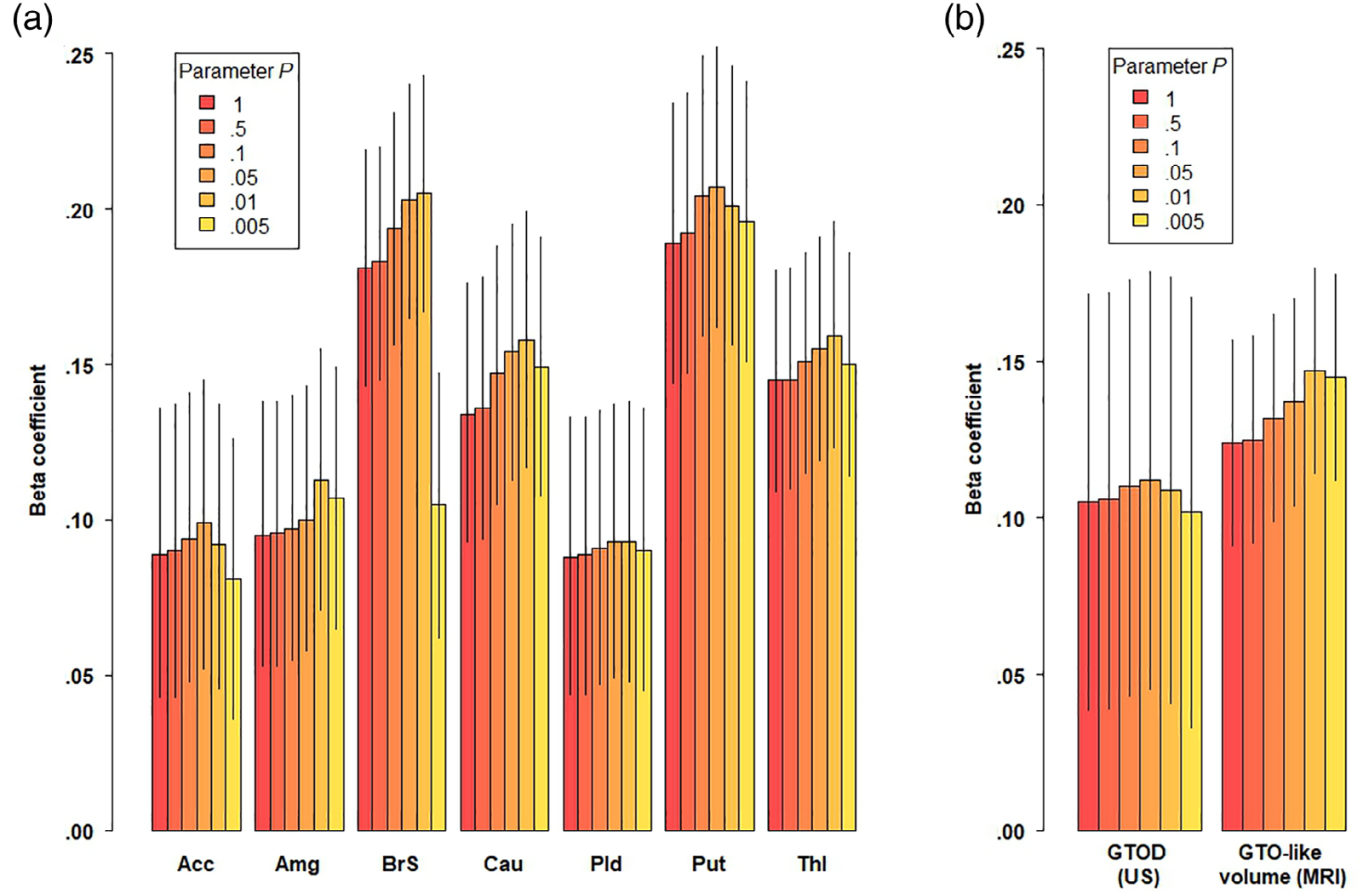
The beta coefficients for the association between subcortical PGS and the brain. The colored bars represent the different values for the P parameter for LDpred. The error bars denote the 95% confidence interval. Panel a shows the results for PGS and their associated regions during early childhood. Panel b shows the associations for the GTO PGS and the corresponding measures from ultrasound and MRI. Acc, accumbens; Amg, amygdala; BrS, brainstem; Cau, caudate nucleus; MRI, magnetic resonance imaging; Pld, pallidus; Put, putamen; Thl, thalamus; US, ultrasound

The volumes of all regions were correlated, with most Pearson's *r* values ranging between 0.4 and 0.6 (Figure S3). The region‐specific associations between the PGS and mean volumes are shown in Figure 3. All regional volumes were significantly associated with their PGS, with the strongest associations in the putamen (*β* = 0.207, 95% CI = [0.162; 0.252], Δ*R* ^2^ = 4.7%) and the brainstem (*β* = 0.205, 95% CI = [0.167; 0.243], ΔR^2^ = 4.5%). Most importantly, the associations for all regions were generally constant across different values for parameter *P*. The interaction term of PGS with sex was not significant in any of the associations. The correlations were similar in the children with only complete data (Figure S4).

**FIGURE 4:**
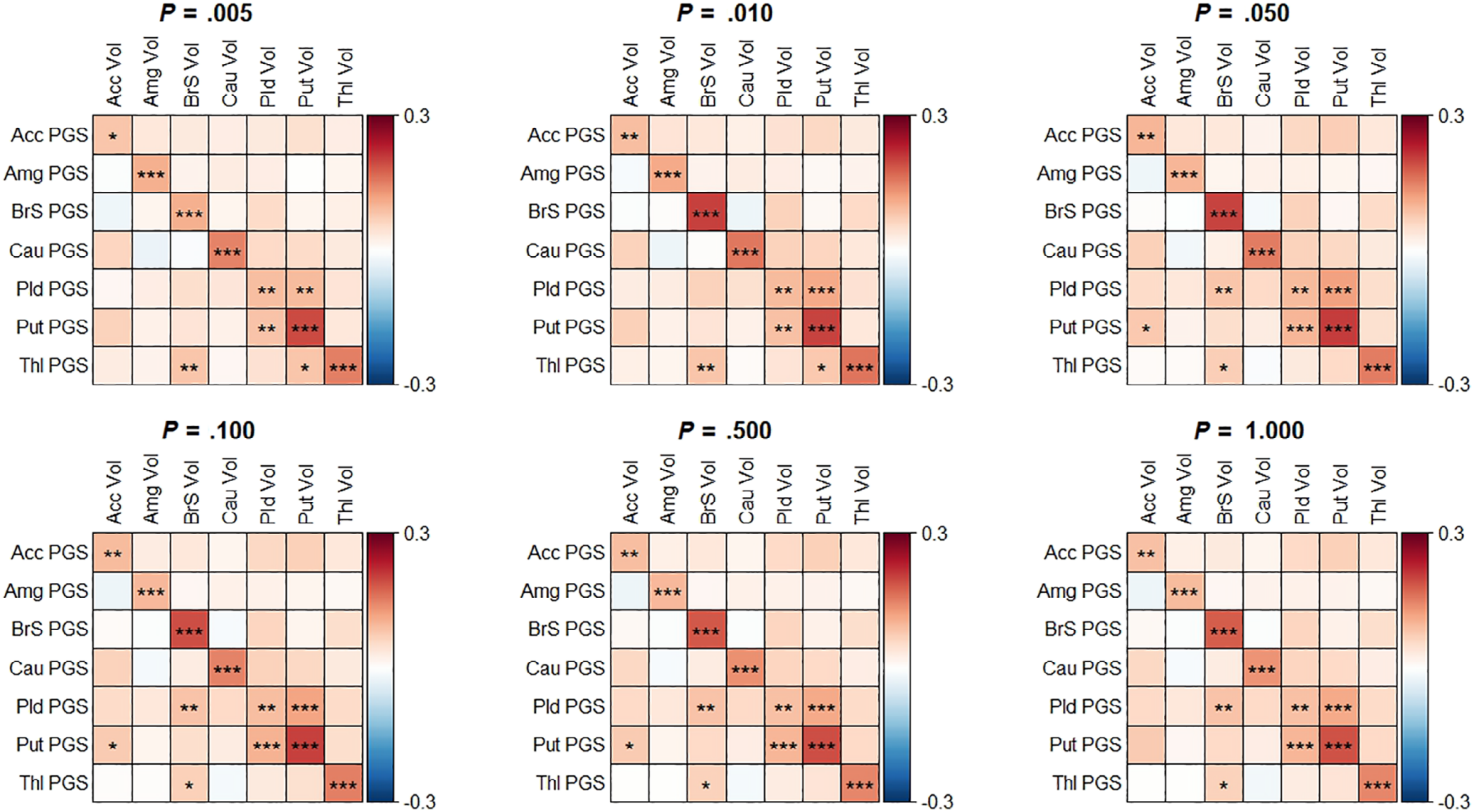
Heatmaps for associations between the subcortical PGS and the MRI‐based subcortical volumes.. Each heatmap corresponds to a different value for parameter P. The rows represent the PGS. Columns represent the standardized volumes for the subcortical regions as obtained from the MRI segmentations. The associations were Bonferroni corrected. Acc, accumbens; Amg, amygdala; BrS, brainstem; Cau, caudate nucleus; Pld, pallidus; Put, putamen; Thl, thalamus; Vol, volume. *p‐value <.05; **p‐value <.01; ***p‐value <.001

Region‐nonspecific associations are shown in Figure 4 and Table S1 for PGS based on all parameters *P*. After Bonferroni correction only 3–4 out of 42 region‐nonspecific associations reached statistical significance across parameter *P* values, compared to 7 out of 7 for the region‐specific associations. Two of the region‐nonspecific associations that were statistically significant for all parameter *P* values were located within the globus pallidus and the putamen (both *p* <.001), which are neighboring regions. Sensitivity analyses showed that the pattern of associations generally remained similar after excluding participants with lower region‐specific segmentation quality and those without segmentation quality ratings (Figure S5). However, the region‐specific association between the PGS and volume of the nucleus accumbens did not reach statistical significance anymore in all but the parameter *P* = .05 level. In children with only complete data only the region‐specific associations were statistically significant, and the accumbens and pallidus associations did not reach statistical significance at any level of parameter *P* (Figure S6). Similar patterns emerged in children with only complete data after excluding those with poor image segmentation quality and those without segmentation quality ratings (Figure S7).

### 3.3 GTO PGS as a predictor for infant US and childhood MRI

We constructed a GTO PGS by combining the scores for the caudate nucleus, the putamen, the globus pallidus and the thalamus. We further combined the volumes of these regions as obtained from the MR images at 10 years to approximate a GTO‐like volume. As Figure 3b and Table S2 show, the GTO PGS at all levels of parameter *P* seemed to associate with similar effect size to the GTOD and the GTO‐like volume from childhood MRI. For example, at parameter *P* = 1.00 one *SD* increase in the PGS led to a 0.105 (95% CI = [0.039; 0.171]) increase in standardized GTOD and 0.124 (95% CI = [0.091; 0.157]) increase in standardized GTO‐like volume.

The GTOD from ultrasound also related to the GTO‐like volume from childhood MRI, with each *SD* increase in GTOD associating with a 0.27 standardized increase in GTO‐like volume (95% CI = [0.17; 0.36], Δ*R* ^2^ = 3.9%).

### 3.4 Mediation analyses

To assess the relative contribution of the GTO PGS to GTOD and the GTO‐like volume we constructed a causal mediation model. The results for the mediation analyses at all parameter *P* values are shown in Table 2. We found that the effect of the combined PGS on the estimated GTO volume at 10 years of age was partly mediated by the GTOD at 7 weeks of age at parameter *P* = .05 and higher. At those thresholds, the natural direct effect ranged between 82.4 and 83.5%, and the natural indirect effect between 16.5 and 17.6%. Substituting the GTO‐like volume for its estimated diameter did not attenuate or change the results, with the proportion of the mediated effect increasing by less than 0.5% for all parameter *P* values (Table S3).

**TABLE 2:** Results for the mediation analyses

Parameter *P*	Direct effect (95% CI)	Indirect effect (95% CI)	*p*‐value
.005	87.4% (67.4–100.0)	12.6% (0.0–32.6)	.134
.010	85.9% (68.4–100.0)	14.1% (0.0–31.6)	.058
.050	83.5% (60.7–97.8)	16.5% (2.2–39.3)	.018
.100	83.0% (61.4–97.7)	17.0% (2.3–38.6)	.028
.500	82.5% (59.8–98.0)	17.5% (2.0–40.2)	.036
1.000	82.4% (58.7–97.5)	17.6% (2.5–41.3)	.028

*Note:* The percentages represent the proportions of the natural direct effect and the natural indirect, that is, mediated, effect.

## 4 DISCUSSION

The genetic loci related to subcortical volumes in an adult sample also associate with subcortical volume during infancy and early childhood. Furthermore, the associations during early childhood are partly independent from those during infancy, suggesting that the previously identified loci affect both prenatal and postnatal development.

The region‐specific PGS primarily associated with the region‐specific volumes from MRI, and had limited associations with other regions. The previously identified genetic loci therefore generalize to earlier life phases, even infancy, and could aid in predictions based on subcortical volumes. The coefficients obtained from the analyses may be considered modest in size. In general, a *SD* change in the PGS only led to about 0.1 *SD* increase in the associated subcortical volume. Interestingly, this effect was similar to the effect size of sex and the effect size of a year increase in age in the early childhood MRI. In addition, PGS tend to have effect sizes around this magnitude (P. R. Jansen et al., 2019). This suggests that the PGS did capture a relevant effect in prediction of subcortical volume in childhood.

The mediation analysis suggested that the genetic effects on subcortical volumes during infancy only explain a relatively small part of the volumes during early childhood. A theoretical consideration is that the analyses were adjusted for global scale, that is, head circumference during infancy and intracranial volume during early childhood, which was also done in the original GWAS (Satizabal et al., 2019). The analyses therefore focus on the relative size of subcortical regions. Combined, this implies that the genetic scores capture a change in the relative subcortical volumes that occurs during early childhood, that is at least partly independent of the volumes during infancy. Little is known, however, about growth trajectories of subcortical volume relative to intracranial volume during early life. A study of 48 postmortem fetal brains found that the deep nuclei—which roughly correspond to the subcortical regions of interest—did not increase in size relative to the supratentorial volume between gestational weeks 20–31 (Scott et al., 2011). By contrast, a study by the ENIGMA consortium showed that the subcortical volumes corrected for intracranial volume rapidly change particularly during early childhood (Dima et al., 2020). Further work is needed to study whether and how trajectories of prenatal subcortical volumes relate to postnatal trajectories, and how the underlying genetic mechanisms differ.

Another explanation for the relatively weak mediating effect relates to individual differences in brain development. Subcortical volumes during early childhood may already relate very closely to the peak volumes as seen during adulthood, given that the brain reaches its maximum size at approximately 10–12 years of age (Giedd, 2004; Shaw et al., 2008). However, individuals reach these end states of development at different rates (Karolis et al., 2017; Raznahan et al., 2014). Individuals who reach the same peak volume may do so at very different ages due to differences in developmental rates during in utero and early childhood. Studying regional brain volumes during infancy may therefore be confounded by differences in developmental trajectories that are perhaps driven by different genetic mechanisms, thus leading to an underestimation of the mediating effect. These differences in developmental trajectories may be amplified in subcortical regions like the thalamus, which develop throughout adolescence and reach their peak volume during adulthood (Wang, Xu, Luo, Hu, & Zuo, 2019). Further work on infant brain development is needed to address these questions.

The study had several limitations. First, the original GWAS were based on MRI data whereas the imaging during infancy was done using cranial ultrasound. These methods have vastly different levels of precision, with the ultrasound potentially suffering from more measurement error compared to the MRI. In addition, the ultrasound data focused on the GTO, which does not have a proper equivalent structure in the MRI analyses and the GWAS. These differences in measurement error and region of interest have likely led to an underestimation of the mediating effect of subcortical volumes during infancy. Second, the exclusion of those genetically classified as non‐European leads to a severe reduction in sample size and further limits the generalizability of the findings. Third, the sample with complete data was only about a quarter of the sample with useable data. We tried to account for this limitation by also showing the analyses for both datasets, and we further showed that the characteristics of the samples and the effect sizes of each model are comparable. Fourth, any residual confounding in the mediation analysis may lead to overestimation of the mediating effect (VanderWeele, 2016). We corrected for educational attainment of the mother and birth weight of the child in the association between infant and early childhood GTO volume, but other confounders could still be unaccounted for. Fifth, the GWAS on subcortical volumes focused on SNPs, which cover only part of the genetic variation between individuals. If all genetic variation underlying subcortical volumes could be captures in a score then the identified associations may have been different. However, given the low prevalence of other sources of genetic variation—like indels and copy‐number variants—it would require massive sample sizes to identify those. Our study also benefitted from a number of strengths. As the sample size was reasonably large, the statistical power was high enough to detect relatively small effects. For example, for the associations between the PGS and early childhood MRI volumes, the a priori power to detect 2% explained variance on top of the base model was 98.1% for unadjusted analyses and 78.8% for the Bonferroni adjusted analyses. Finally, the Generation R study provided a unique sample with prospectively collected neuroimaging during infancy and childhood.

In conclusion, we established that genetic variants related to subcortical volume during adulthood also relate to subcortical volume during infancy and early childhood. We further showed that these effects are partly independent, suggesting that the genetic loci may modulate specific stages of postnatal development.

## CONFLICT OF INTEREST

The authors declare no conflict of interest.

## DATA AVAILABILITY STATEMENT

The data used in this study will not be made publicly available due to legal and informed consent restrictions. Reasonable requests for access to the data can be directed to the Director of the Generation R Study, Vincent Jaddoe (generationr@erasmusmc.nl), in accordance with the local, national and European Union regulations.

## Supporting information


**Figure S1** Correlation matrix for the PGSs in the children with MRI data (*n* = 1,201). Pearson's *r* values are reported. The numbers represent the different values for the *P* parameter for LDpred. The GTO‐like PGS was calculated by combining the PGS for the caudate nuclei, the pallidi, the putamen and the thalami. Acc, accumbens; Amg, amygdala; BrS, brainstem; Cau, caudate nucleus; GTO, gangliothalamic ovoid; Pld, pallidus; Put, putamen; Thl, thalamus.

**Figure S2** Correlation matrix for the PGSs in the children with complete data (*n* = 340). Pearson's *r* values are reported. The numbers represent the different values for the *P* parameter for LDpred. The GTO‐like PGS was calculated by combining the PGS for the caudate nuclei, the pallidi, the putamen and the thalami. Acc, accumbens; Amg, amygdala; BrS, brainstem; Cau, caudate nucleus; GTO, gangliothalamic ovoid; Pld, pallidus; Put, putamen; Thl, thalamus.

**Figure S3** Correlation matrix for MRI‐based subcortical volumes. Pearson's *r* values are reported. Acc, accumbens; Amg, amygdala; BrS, brainstem; Cau, caudate nucleus; Pld, pallidus; Put, putamen; Thl, thalamus.

**Figure S4** Correlation matrix for MRI‐based subcortical volumes, the MRI GTO‐like volume and the GTOD from the ultrasound, in children with complete data. Pearson's *r* values are reported. The GTO‐like volume for the childhood MRI was calculated by combining the volumes of the caudate nuclei, the pallidi, the putamen and the thalami. Acc, accumbens; Amg, amygdala; BrS, brainstem; Cau, caudate nucleus; GTO, gangliothalamic ovoid; GTOD, GTO distance; Pld, pallidus; Put, putamen; Thl, thalamus.

**Figure S5** Heatmaps for associations between the subcortical PGS and the MRI‐based subcortical volumes, after excluding children with poor segmentation quality. Each heatmap corresponds to a different value for parameter *P*. The rows represent the PGS. Columns represent the standardized volumes for the subcortical regions as obtained from the MRI segmentations. The associations were Bonferroni corrected. Acc, accumbens; Amg, amygdala; BrS, brainstem; Cau, caudate nucleus; Pld, pallidus; Put, putamen; Thl, thalamus; Vol, volume.

**Figure S6** Heatmaps for associations between the subcortical PGS and the MRI‐based subcortical volumes, in children with complete data (*n* = 340). Each heatmap corresponds to a different value for parameter *P*. The rows represent the PGS. Columns represent the standardized volumes for the subcortical regions as obtained from the MRI segmentations. The associations were Bonferroni corrected. Acc, accumbens; Amg, amygdala; BrS, brainstem; Cau, caudate nucleus; Pld, pallidus; Put, putamen; Thl, thalamus; Vol, volume.

**Figure S7** Heatmaps for associations between the subcortical PGS and the MRI‐based subcortical volumes, in children with complete data, after excluding children with poor segmentation quality and those without ratings. Each heatmap corresponds to a different value for parameter *P*. The rows represent the PGS. Columns represent the standardized volumes for the subcortical regions as obtained from the MRI segmentations. The associations were Bonferroni corrected. Acc, accumbens; Amg, amygdala; BrS, brainstem; Cau, caudate nucleus; Pld, pallidus; Put, putamen; Thl, thalamus; Vol, volume.

**Table S1** Full results for the associations between the subcortical PGS and the MRI‐based subcortical volumes. The parameter *P* values are given between the parentheses in the determinant column.

**Table S2** Full results for the associations of the GTO PGS with the GTOD from ultrasound and the MRI‐based GTO‐like volume.

**Table S3** Mediation results for the mediation models where the GTO‐like volume is replaced with an estimated diameter based on a spherical model.
