# Supplementary figures and images for: Genetic scores for adult subcortical volumes associate with subcortical volumes during infancy and childhood
Source: Hum Brain Mapp. 2021 Feb 2;42(6):1583–93. doi: 10.1002/hbm.25292 (PMC7978120; doi:10.1002/hbm.25292)

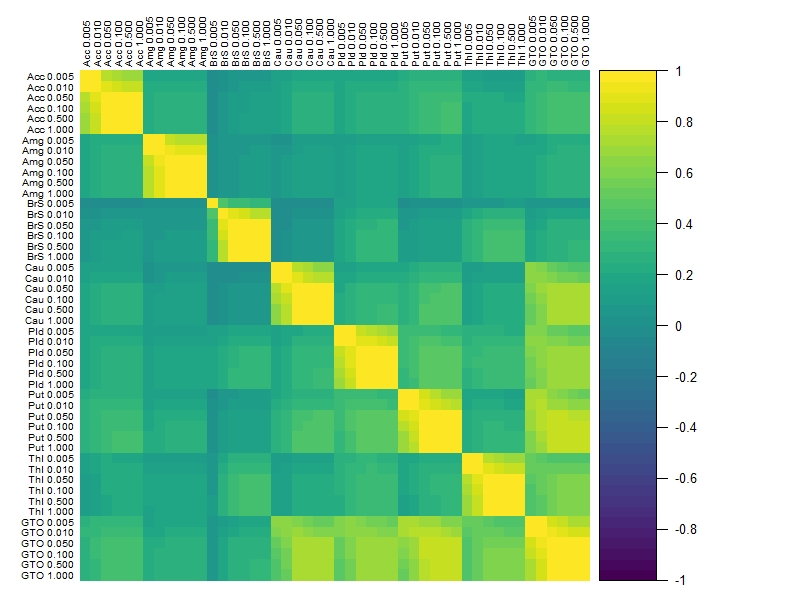

Supplement: Supplementary file 1 — Figure S1 Correlation matrix for the PGSs in the children with MRI data (n = 1,201). Pearson's r values are reported. The numbers represent the different values for the P parameter for LDpred. The GTO‐like PGS was calculated by combining the PGS for the caudate nuclei, the pallidi, the putamen and the thalami. Acc, accumbens; Amg, amygdala; BrS, brainstem; Cau, caudate nucleus; GTO, gangliothalamic ovoid; Pld, pallidus; Put, putamen; Thl, thalamus. [file HBM-42-1583-s003.tiff]

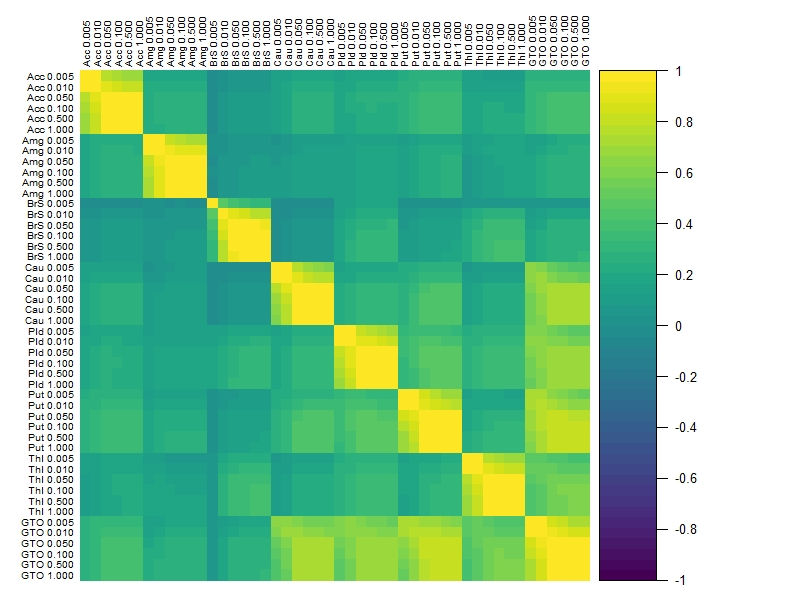

Supplement: Supplementary file 2 — Figure S2 Correlation matrix for the PGSs in the children with complete data (n = 340). Pearson's r values are reported. The numbers represent the different values for the P parameter for LDpred. The GTO‐like PGS was calculated by combining the PGS for the caudate nuclei, the pallidi, the putamen and the thalami. Acc, accumbens; Amg, amygdala; BrS, brainstem; Cau, caudate nucleus; GTO, gangliothalamic ovoid; Pld, pallidus; Put, putamen; Thl, thalamus. [file HBM-42-1583-s007.tiff]

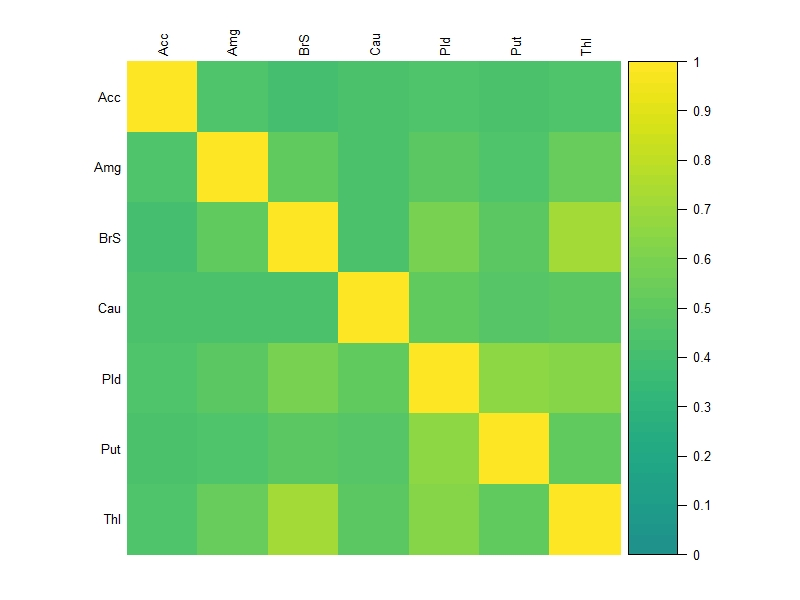

Supplement: Supplementary file 3 — Figure S3 Correlation matrix for MRI‐based subcortical volumes. Pearson's r values are reported. Acc, accumbens; Amg, amygdala; BrS, brainstem; Cau, caudate nucleus; Pld, pallidus; Put, putamen; Thl, thalamus. [file HBM-42-1583-s009.tiff]

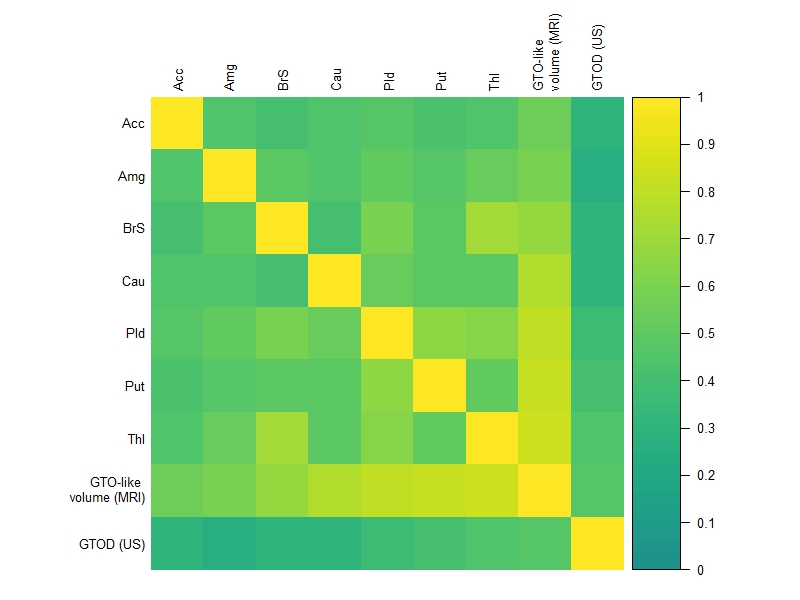

Supplement: Supplementary file 4 — Figure S4 Correlation matrix for MRI‐based subcortical volumes, the MRI GTO‐like volume and the GTOD from the ultrasound, in children with complete data. Pearson's r values are reported. The GTO‐like volume for the childhood MRI was calculated by combining the volumes of the caudate nuclei, the pallidi, the putamen and the thalami. Acc, accumbens; Amg, amygdala; BrS, brainstem; Cau, caudate nucleus; GTO, gangliothalamic ovoid; GTOD, GTO distance; Pld, pallidus; Put, putamen; Thl, thalamus. [file HBM-42-1583-s004.tiff]

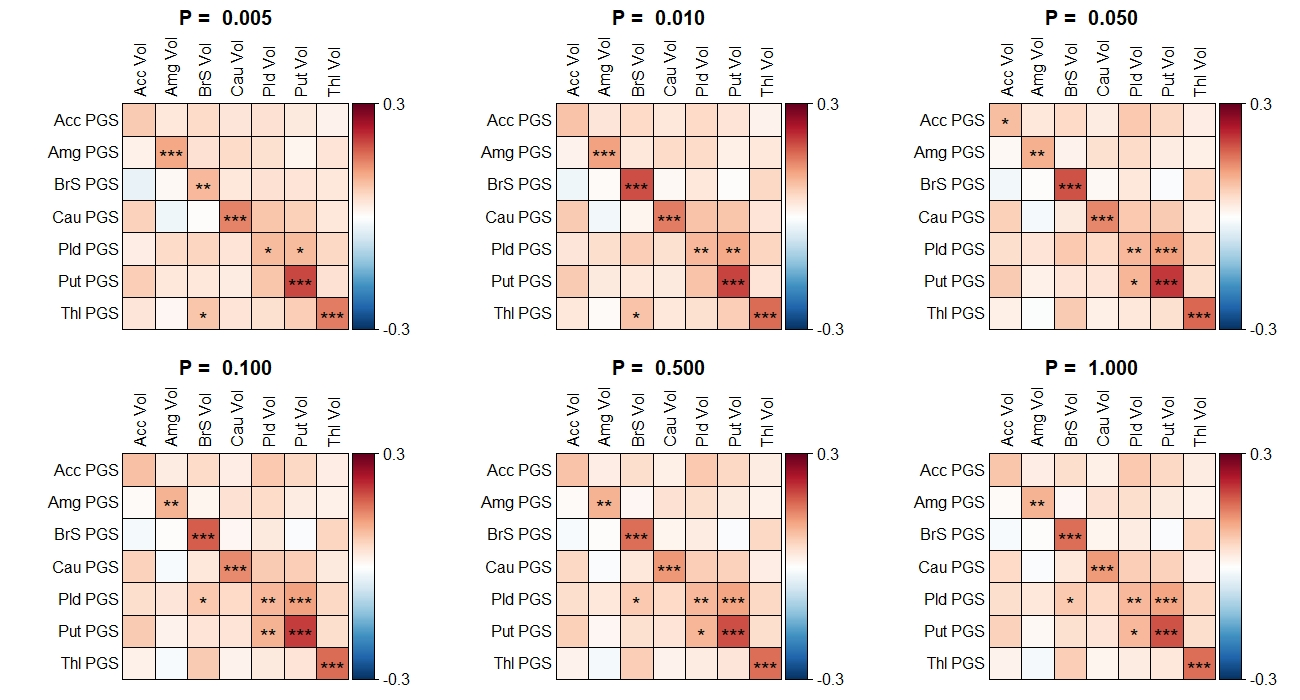

Supplement: Supplementary file 5 — Figure S5 Heatmaps for associations between the subcortical PGS and the MRI‐based subcortical volumes, after excluding children with poor segmentation quality. Each heatmap corresponds to a different value for parameter P. The rows represent the PGS. Columns represent the standardized volumes for the subcortical regions as obtained from the MRI segmentations. The associations were Bonferroni corrected. Acc, accumbens; Amg, amygdala; BrS, brainstem; Cau, caudate nucleus; Pld, pallidus; Put, putamen; Thl, thalamus; Vol, volume. [file HBM-42-1583-s002.tiff]

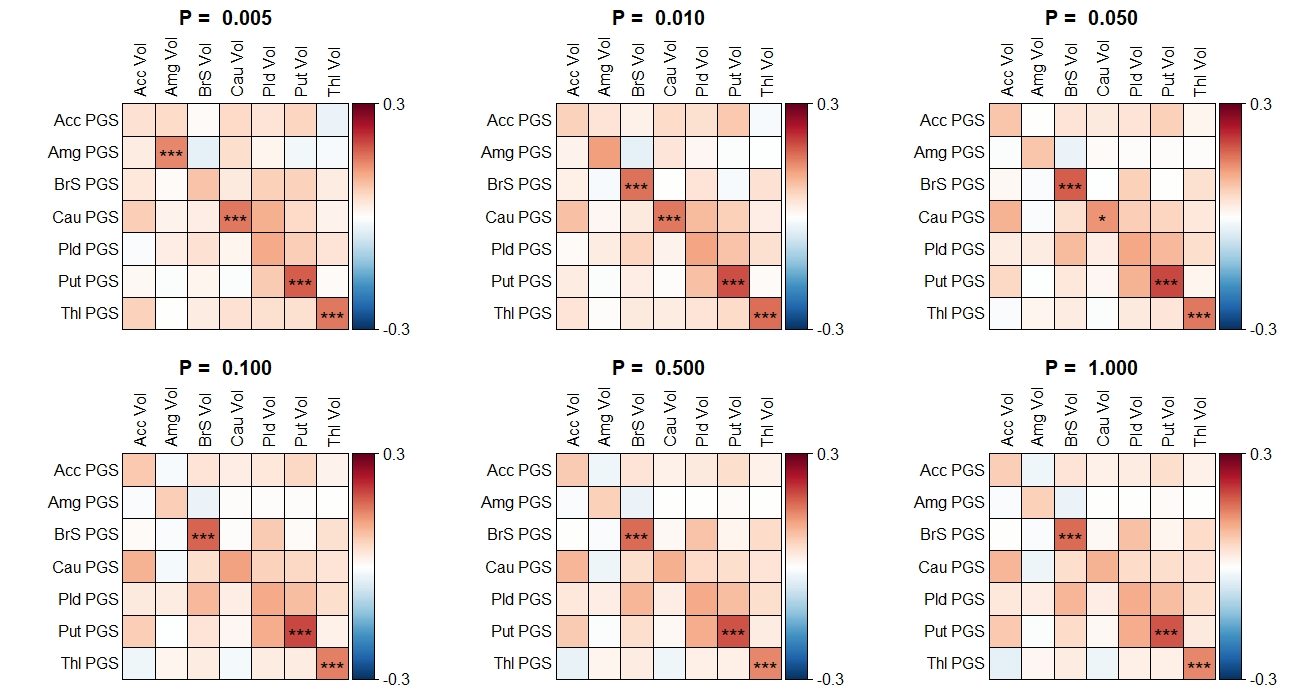

Supplement: Supplementary file 6 — Figure S6 Heatmaps for associations between the subcortical PGS and the MRI‐based subcortical volumes, in children with complete data (n = 340). Each heatmap corresponds to a different value for parameter P. The rows represent the PGS. Columns represent the standardized volumes for the subcortical regions as obtained from the MRI segmentations. The associations were Bonferroni corrected. Acc, accumbens; Amg, amygdala; BrS, brainstem; Cau, caudate nucleus; Pld, pallidus; Put, putamen; Thl, thalamus; Vol, volume. [file HBM-42-1583-s008.tiff]

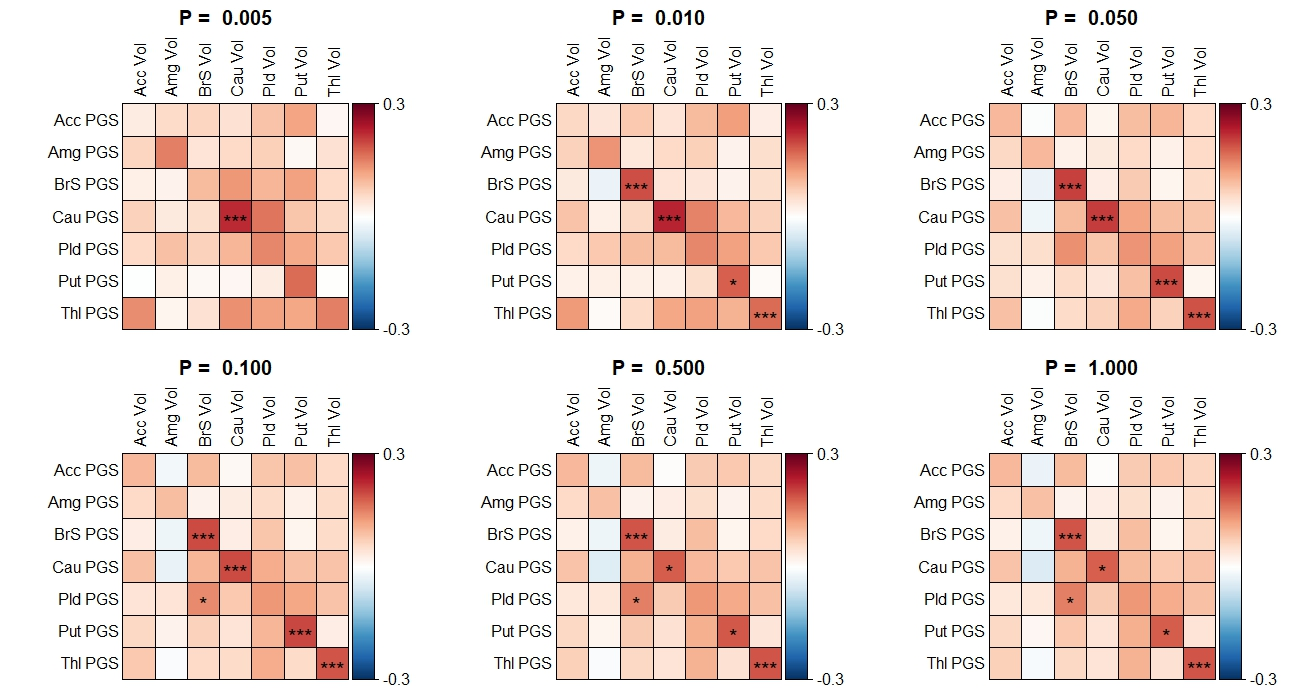

Supplement: Supplementary file 7 — Figure S7 Heatmaps for associations between the subcortical PGS and the MRI‐based subcortical volumes, in children with complete data, after excluding children with poor segmentation quality and those without ratings. Each heatmap corresponds to a different value for parameter P. The rows represent the PGS. Columns represent the standardized volumes for the subcortical regions as obtained from the MRI segmentations. The associations were Bonferroni corrected. Acc, accumbens; Amg, amygdala; BrS, brainstem; Cau, caudate nucleus; Pld, pallidus; Put, putamen; Thl, thalamus; Vol, volume. [file HBM-42-1583-s010.tiff]
