# Supplementary material for: Genetic scores for adult subcortical volumes associate with subcortical volumes during infancy and childhood
Source: Hum Brain Mapp. 2021 Feb 2;42(6):1583–93. doi: 10.1002/hbm.25292 (PMC7978120; doi:10.1002/hbm.25292)
Supplement: Supplementary file 8 — Table S1 Full results for the associations between the subcortical PGS and the MRI‐based subcortical volumes. The parameter P values are given between the parentheses in the determinant column. [file HBM-42-1583-s006.docx]

**Supplementary Table 1. Full results for the associations between the subcortical PGS and the MRI-based subcortical volumes.** The parameter *P* values are given between the parentheses in the determinant column.

| **Determinant** | **Outcome** | **Beta** | **95% CI (lower)** | **95% CI (upper)** | **p-value** | **R2 baseline (%)** | **R2 change (%)** |
| --- | --- | --- | --- | --- | --- | --- | --- |
| Accumbens PGS (0.005) | Accumbens (MRI) | 0.081 | 0.036 | 0.126 | 0.000458824 | 26.40 | 0.80 |
| Amygdala PGS (0.005) | Accumbens (MRI) | -0.005 | -0.050 | 0.040 | 0.828106066 | 25.60 | 0.00 |
| Brainstem PGS (0.005) | Accumbens (MRI) | -0.021 | -0.070 | 0.028 | 0.404413278 | 25.60 | 0.00 |
| Caudate PGS (0.005) | Accumbens (MRI) | 0.065 | 0.020 | 0.109 | 0.004180332 | 26.10 | 0.50 |
| Pallidum PGS (0.005) | Accumbens (MRI) | 0.014 | -0.033 | 0.061 | 0.557238991 | 25.60 | 0.00 |
| Putamen PGS (0.005) | Accumbens (MRI) | 0.067 | 0.022 | 0.112 | 0.003691204 | 26.10 | 0.50 |
| Thalamus PGS (0.005) | Accumbens (MRI) | 0.031 | -0.015 | 0.077 | 0.184228741 | 25.70 | 0.10 |
| Accumbens PGS (0.005) | Amygdala (MRI) | 0.037 | -0.006 | 0.080 | 0.089024858 | 41.00 | 0.10 |
| Amygdala PGS (0.005) | Amygdala (MRI) | 0.107 | 0.065 | 0.149 | 7.11788E-07 | 42.10 | 1.20 |
| Brainstem PGS (0.005) | Amygdala (MRI) | 0.017 | -0.029 | 0.064 | 0.46202431 | 40.90 | 0.00 |
| Caudate PGS (0.005) | Amygdala (MRI) | -0.026 | -0.067 | 0.016 | 0.230279716 | 40.90 | 0.10 |
| Pallidum PGS (0.005) | Amygdala (MRI) | 0.027 | -0.018 | 0.071 | 0.236179394 | 40.90 | 0.10 |
| Putamen PGS (0.005) | Amygdala (MRI) | 0.022 | -0.021 | 0.065 | 0.319480861 | 40.90 | 0.00 |
| Thalamus PGS (0.005) | Amygdala (MRI) | 0.019 | -0.024 | 0.063 | 0.387105524 | 40.90 | 0.00 |
| Accumbens PGS (0.005) | Brainstem (MRI) | 0.035 | -0.005 | 0.074 | 0.085181793 | 49.10 | 0.10 |
| Amygdala PGS (0.005) | Brainstem (MRI) | 0.018 | -0.021 | 0.057 | 0.373990047 | 49.00 | 0.00 |
| Brainstem PGS (0.005) | Brainstem (MRI) | 0.105 | 0.062 | 0.147 | 1.61523E-06 | 50.00 | 1.00 |
| Caudate PGS (0.005) | Brainstem (MRI) | -0.007 | -0.045 | 0.032 | 0.740602558 | 49.00 | 0.00 |
| Pallidum PGS (0.005) | Brainstem (MRI) | 0.053 | 0.013 | 0.094 | 0.010299919 | 49.30 | 0.30 |
| Putamen PGS (0.005) | Brainstem (MRI) | 0.043 | 0.004 | 0.083 | 0.031892089 | 49.20 | 0.20 |
| Thalamus PGS (0.005) | Brainstem (MRI) | 0.082 | 0.042 | 0.122 | 5.44601E-05 | 49.70 | 0.70 |
| Accumbens PGS (0.005) | Caudate (MRI) | 0.028 | -0.015 | 0.071 | 0.2062411 | 32.70 | 0.10 |
| Amygdala PGS (0.005) | Caudate (MRI) | 0.031 | -0.012 | 0.073 | 0.155804408 | 32.70 | 0.10 |
| Brainstem PGS (0.005) | Caudate (MRI) | 0.015 | -0.032 | 0.061 | 0.540052253 | 32.60 | 0.00 |
| Caudate PGS (0.005) | Caudate (MRI) | 0.149 | 0.108 | 0.191 | 1.65716E-12 | 35.40 | 2.80 |
| Pallidum PGS (0.005) | Caudate (MRI) | 0.040 | -0.005 | 0.084 | 0.07839729 | 32.80 | 0.20 |
| Putamen PGS (0.005) | Caudate (MRI) | 0.021 | -0.022 | 0.064 | 0.33904388 | 32.70 | 0.10 |
| Thalamus PGS (0.005) | Caudate (MRI) | 0.014 | -0.030 | 0.057 | 0.537436394 | 32.60 | 0.00 |
| Accumbens PGS (0.005) | Pallidum (MRI) | 0.035 | -0.010 | 0.079 | 0.124435959 | 33.40 | 0.10 |
| Amygdala PGS (0.005) | Pallidum (MRI) | 0.027 | -0.017 | 0.071 | 0.22960204 | 33.40 | 0.10 |
| Brainstem PGS (0.005) | Pallidum (MRI) | 0.055 | 0.006 | 0.103 | 0.02611354 | 33.60 | 0.30 |
| Caudate PGS (0.005) | Pallidum (MRI) | 0.058 | 0.015 | 0.101 | 0.007945078 | 33.70 | 0.40 |
| Pallidum PGS (0.005) | Pallidum (MRI) | 0.090 | 0.045 | 0.136 | 0.000103452 | 34.10 | 0.90 |
| Putamen PGS (0.005) | Pallidum (MRI) | 0.086 | 0.042 | 0.130 | 0.000145498 | 34.10 | 0.80 |
| Thalamus PGS (0.005) | Pallidum (MRI) | 0.047 | 0.002 | 0.092 | 0.040491055 | 33.50 | 0.20 |
| Accumbens PGS (0.005) | Putamen (MRI) | 0.049 | 0.003 | 0.095 | 0.036577796 | 28.10 | 0.30 |
| Amygdala PGS (0.005) | Putamen (MRI) | 0.001 | -0.044 | 0.047 | 0.950404214 | 27.80 | 0.00 |
| Brainstem PGS (0.005) | Putamen (MRI) | 0.017 | -0.033 | 0.068 | 0.501885974 | 27.90 | 0.00 |
| Caudate PGS (0.005) | Putamen (MRI) | 0.054 | 0.009 | 0.100 | 0.017898475 | 28.20 | 0.30 |
| Pallidum PGS (0.005) | Putamen (MRI) | 0.095 | 0.047 | 0.142 | 9.74582E-05 | 28.80 | 0.90 |
| Putamen PGS (0.005) | Putamen (MRI) | 0.196 | 0.151 | 0.241 | 3.70642E-17 | 32.10 | 4.20 |
| Thalamus PGS (0.005) | Putamen (MRI) | 0.081 | 0.035 | 0.128 | 0.000646819 | 28.60 | 0.70 |
| Accumbens PGS (0.005) | Thalamus (MRI) | 0.028 | -0.009 | 0.064 | 0.139545774 | 55.90 | 0.10 |
| Amygdala PGS (0.005) | Thalamus (MRI) | 0.017 | -0.019 | 0.054 | 0.347382786 | 55.90 | 0.00 |
| Brainstem PGS (0.005) | Thalamus (MRI) | 0.025 | -0.015 | 0.065 | 0.218032676 | 55.90 | 0.10 |
| Caudate PGS (0.005) | Thalamus (MRI) | 0.033 | -0.003 | 0.069 | 0.073862358 | 56.00 | 0.10 |
| Pallidum PGS (0.005) | Thalamus (MRI) | 0.042 | 0.004 | 0.080 | 0.031954004 | 56.00 | 0.20 |
| Putamen PGS (0.005) | Thalamus (MRI) | 0.037 | 0.000 | 0.074 | 0.049610486 | 56.00 | 0.10 |
| Thalamus PGS (0.005) | Thalamus (MRI) | 0.150 | 0.114 | 0.186 | 1.52004E-15 | 58.20 | 2.30 |
| Accumbens PGS (0.010) | Accumbens (MRI) | 0.092 | 0.046 | 0.137 | 7.69932E-05 | 26.60 | 1.00 |
| Amygdala PGS (0.010) | Accumbens (MRI) | -0.012 | -0.057 | 0.033 | 0.610473611 | 25.60 | 0.00 |
| Brainstem PGS (0.010) | Accumbens (MRI) | -0.004 | -0.049 | 0.041 | 0.857316864 | 25.60 | 0.00 |
| Caudate PGS (0.010) | Accumbens (MRI) | 0.068 | 0.024 | 0.112 | 0.002501031 | 26.20 | 0.60 |
| Pallidum PGS (0.010) | Accumbens (MRI) | 0.032 | -0.014 | 0.079 | 0.169385864 | 25.70 | 0.10 |
| Putamen PGS (0.010) | Accumbens (MRI) | 0.071 | 0.025 | 0.116 | 0.002234767 | 26.20 | 0.60 |
| Thalamus PGS (0.010) | Accumbens (MRI) | 0.026 | -0.020 | 0.071 | 0.27406307 | 25.70 | 0.10 |
| Accumbens PGS (0.010) | Amygdala (MRI) | 0.043 | 0.000 | 0.085 | 0.052222109 | 41.10 | 0.20 |
| Amygdala PGS (0.010) | Amygdala (MRI) | 0.113 | 0.071 | 0.155 | 1.59109E-07 | 42.20 | 1.40 |
| Brainstem PGS (0.010) | Amygdala (MRI) | 0.003 | -0.040 | 0.045 | 0.901113751 | 40.90 | 0.00 |
| Caudate PGS (0.010) | Amygdala (MRI) | -0.024 | -0.066 | 0.018 | 0.25876963 | 40.90 | 0.10 |
| Pallidum PGS (0.010) | Amygdala (MRI) | 0.031 | -0.012 | 0.075 | 0.159794989 | 41.00 | 0.10 |
| Putamen PGS (0.010) | Amygdala (MRI) | 0.021 | -0.021 | 0.064 | 0.326958116 | 40.90 | 0.00 |
| Thalamus PGS (0.010) | Amygdala (MRI) | 0.016 | -0.027 | 0.059 | 0.472384401 | 40.90 | 0.00 |
| Accumbens PGS (0.010) | Brainstem (MRI) | 0.037 | -0.002 | 0.077 | 0.065650835 | 49.20 | 0.10 |
| Amygdala PGS (0.010) | Brainstem (MRI) | 0.019 | -0.021 | 0.058 | 0.350227454 | 49.00 | 0.00 |
| Brainstem PGS (0.010) | Brainstem (MRI) | 0.205 | 0.167 | 0.243 | 1.43931E-25 | 53.50 | 4.50 |
| Caudate PGS (0.010) | Brainstem (MRI) | 0.005 | -0.033 | 0.044 | 0.791054127 | 49.00 | 0.00 |
| Pallidum PGS (0.010) | Brainstem (MRI) | 0.067 | 0.027 | 0.107 | 0.001155302 | 49.50 | 0.50 |
| Putamen PGS (0.010) | Brainstem (MRI) | 0.045 | 0.006 | 0.085 | 0.02528497 | 49.20 | 0.20 |
| Thalamus PGS (0.010) | Brainstem (MRI) | 0.083 | 0.044 | 0.123 | 4.09702E-05 | 49.70 | 0.70 |
| Accumbens PGS (0.010) | Caudate (MRI) | 0.026 | -0.017 | 0.070 | 0.230131591 | 32.70 | 0.10 |
| Amygdala PGS (0.010) | Caudate (MRI) | 0.030 | -0.013 | 0.073 | 0.166140984 | 32.70 | 0.10 |
| Brainstem PGS (0.010) | Caudate (MRI) | -0.022 | -0.065 | 0.021 | 0.308118379 | 32.70 | 0.10 |
| Caudate PGS (0.010) | Caudate (MRI) | 0.158 | 0.117 | 0.199 | 7.31392E-14 | 35.70 | 3.10 |
| Pallidum PGS (0.010) | Caudate (MRI) | 0.049 | 0.005 | 0.093 | 0.028678801 | 32.90 | 0.30 |
| Putamen PGS (0.010) | Caudate (MRI) | 0.023 | -0.020 | 0.066 | 0.295691107 | 32.70 | 0.10 |
| Thalamus PGS (0.010) | Caudate (MRI) | 0.007 | -0.036 | 0.050 | 0.74949102 | 32.60 | 0.00 |
| Accumbens PGS (0.010) | Pallidum (MRI) | 0.046 | 0.002 | 0.090 | 0.042018281 | 33.50 | 0.20 |
| Amygdala PGS (0.010) | Pallidum (MRI) | 0.028 | -0.016 | 0.072 | 0.213385934 | 33.40 | 0.10 |
| Brainstem PGS (0.010) | Pallidum (MRI) | 0.068 | 0.024 | 0.112 | 0.002425508 | 33.80 | 0.50 |
| Caudate PGS (0.010) | Pallidum (MRI) | 0.066 | 0.023 | 0.109 | 0.00285022 | 33.80 | 0.50 |
| Pallidum PGS (0.010) | Pallidum (MRI) | 0.093 | 0.048 | 0.138 | 4.99355E-05 | 34.20 | 0.90 |
| Putamen PGS (0.010) | Pallidum (MRI) | 0.088 | 0.044 | 0.133 | 8.56391E-05 | 34.10 | 0.90 |
| Thalamus PGS (0.010) | Pallidum (MRI) | 0.045 | 0.001 | 0.090 | 0.045730987 | 33.50 | 0.20 |
| Accumbens PGS (0.010) | Putamen (MRI) | 0.062 | 0.016 | 0.109 | 0.008606386 | 28.30 | 0.40 |
| Amygdala PGS (0.010) | Putamen (MRI) | 0.010 | -0.036 | 0.056 | 0.670933084 | 27.90 | 0.00 |
| Brainstem PGS (0.010) | Putamen (MRI) | 0.009 | -0.037 | 0.055 | 0.698492813 | 27.90 | 0.00 |
| Caudate PGS (0.010) | Putamen (MRI) | 0.064 | 0.019 | 0.109 | 0.005396661 | 28.30 | 0.50 |
| Pallidum PGS (0.010) | Putamen (MRI) | 0.111 | 0.064 | 0.157 | 4.14822E-06 | 29.10 | 1.30 |
| Putamen PGS (0.010) | Putamen (MRI) | 0.201 | 0.156 | 0.246 | 6.60193E-18 | 32.30 | 4.40 |
| Thalamus PGS (0.010) | Putamen (MRI) | 0.080 | 0.034 | 0.127 | 0.000737334 | 28.50 | 0.70 |
| Accumbens PGS (0.010) | Thalamus (MRI) | 0.033 | -0.004 | 0.070 | 0.081356888 | 56.00 | 0.10 |
| Amygdala PGS (0.010) | Thalamus (MRI) | 0.018 | -0.018 | 0.055 | 0.327778676 | 55.90 | 0.00 |
| Brainstem PGS (0.010) | Thalamus (MRI) | 0.059 | 0.022 | 0.095 | 0.001665849 | 56.20 | 0.40 |
| Caudate PGS (0.010) | Thalamus (MRI) | 0.037 | 0.001 | 0.073 | 0.045011546 | 56.00 | 0.20 |
| Pallidum PGS (0.010) | Thalamus (MRI) | 0.048 | 0.010 | 0.085 | 0.012796099 | 56.10 | 0.20 |
| Putamen PGS (0.010) | Thalamus (MRI) | 0.039 | 0.002 | 0.076 | 0.038446195 | 56.00 | 0.20 |
| Thalamus PGS (0.010) | Thalamus (MRI) | 0.159 | 0.123 | 0.196 | 1.28676E-17 | 58.50 | 2.70 |
| Accumbens PGS (0.050) | Accumbens (MRI) | 0.099 | 0.052 | 0.145 | 2.98563E-05 | 26.70 | 1.10 |
| Amygdala PGS (0.050) | Accumbens (MRI) | -0.021 | -0.067 | 0.024 | 0.360022057 | 25.60 | 0.10 |
| Brainstem PGS (0.050) | Accumbens (MRI) | 0.005 | -0.040 | 0.050 | 0.819601743 | 25.60 | 0.00 |
| Caudate PGS (0.050) | Accumbens (MRI) | 0.071 | 0.026 | 0.115 | 0.001880999 | 26.20 | 0.60 |
| Pallidum PGS (0.050) | Accumbens (MRI) | 0.055 | 0.009 | 0.101 | 0.018095048 | 25.90 | 0.40 |
| Putamen PGS (0.050) | Accumbens (MRI) | 0.078 | 0.033 | 0.123 | 0.000742404 | 26.30 | 0.70 |
| Thalamus PGS (0.050) | Accumbens (MRI) | 0.008 | -0.037 | 0.053 | 0.733858804 | 25.60 | 0.00 |
| Accumbens PGS (0.050) | Amygdala (MRI) | 0.036 | -0.008 | 0.080 | 0.106771931 | 41.00 | 0.10 |
| Amygdala PGS (0.050) | Amygdala (MRI) | 0.100 | 0.058 | 0.143 | 4.00059E-06 | 41.90 | 1.10 |
| Brainstem PGS (0.050) | Amygdala (MRI) | -0.003 | -0.045 | 0.039 | 0.892101094 | 40.90 | 0.00 |
| Caudate PGS (0.050) | Amygdala (MRI) | -0.016 | -0.058 | 0.026 | 0.459457608 | 40.90 | 0.00 |
| Pallidum PGS (0.050) | Amygdala (MRI) | 0.038 | -0.005 | 0.081 | 0.08308689 | 41.00 | 0.20 |
| Putamen PGS (0.050) | Amygdala (MRI) | 0.018 | -0.025 | 0.061 | 0.408814868 | 40.90 | 0.00 |
| Thalamus PGS (0.050) | Amygdala (MRI) | 0.008 | -0.035 | 0.051 | 0.706546858 | 40.90 | 0.00 |
| Accumbens PGS (0.050) | Brainstem (MRI) | 0.037 | -0.004 | 0.077 | 0.076470563 | 49.10 | 0.10 |
| Amygdala PGS (0.050) | Brainstem (MRI) | 0.015 | -0.025 | 0.054 | 0.465194587 | 49.00 | 0.00 |
| Brainstem PGS (0.050) | Brainstem (MRI) | 0.203 | 0.165 | 0.240 | 1.9868E-25 | 53.50 | 4.50 |
| Caudate PGS (0.050) | Brainstem (MRI) | 0.026 | -0.013 | 0.065 | 0.190637038 | 49.10 | 0.10 |
| Pallidum PGS (0.050) | Brainstem (MRI) | 0.083 | 0.043 | 0.122 | 4.46942E-05 | 49.70 | 0.70 |
| Putamen PGS (0.050) | Brainstem (MRI) | 0.051 | 0.011 | 0.090 | 0.011550047 | 49.30 | 0.30 |
| Thalamus PGS (0.050) | Brainstem (MRI) | 0.073 | 0.034 | 0.112 | 0.000263421 | 49.60 | 0.60 |
| Accumbens PGS (0.050) | Caudate (MRI) | 0.020 | -0.024 | 0.064 | 0.372648282 | 32.70 | 0.00 |
| Amygdala PGS (0.050) | Caudate (MRI) | 0.023 | -0.020 | 0.066 | 0.287479236 | 32.70 | 0.10 |
| Brainstem PGS (0.050) | Caudate (MRI) | -0.015 | -0.058 | 0.027 | 0.48343067 | 32.60 | 0.00 |
| Caudate PGS (0.050) | Caudate (MRI) | 0.154 | 0.113 | 0.195 | 4.85558E-13 | 35.50 | 2.90 |
| Pallidum PGS (0.050) | Caudate (MRI) | 0.059 | 0.015 | 0.102 | 0.00810947 | 33.00 | 0.40 |
| Putamen PGS (0.050) | Caudate (MRI) | 0.032 | -0.012 | 0.075 | 0.151376277 | 32.70 | 0.10 |
| Thalamus PGS (0.050) | Caudate (MRI) | -0.009 | -0.052 | 0.034 | 0.689532462 | 32.60 | 0.00 |
| Accumbens PGS (0.050) | Pallidum (MRI) | 0.061 | 0.016 | 0.106 | 0.008472312 | 33.70 | 0.40 |
| Amygdala PGS (0.050) | Pallidum (MRI) | 0.024 | -0.021 | 0.068 | 0.293621782 | 33.30 | 0.10 |
| Brainstem PGS (0.050) | Pallidum (MRI) | 0.067 | 0.024 | 0.111 | 0.002563659 | 33.80 | 0.50 |
| Caudate PGS (0.050) | Pallidum (MRI) | 0.068 | 0.024 | 0.111 | 0.002274952 | 33.80 | 0.50 |
| Pallidum PGS (0.050) | Pallidum (MRI) | 0.093 | 0.049 | 0.137 | 4.06235E-05 | 34.20 | 0.90 |
| Putamen PGS (0.050) | Pallidum (MRI) | 0.101 | 0.057 | 0.145 | 6.89935E-06 | 34.40 | 1.10 |
| Thalamus PGS (0.050) | Pallidum (MRI) | 0.035 | -0.009 | 0.079 | 0.119355679 | 33.40 | 0.10 |
| Accumbens PGS (0.050) | Putamen (MRI) | 0.073 | 0.025 | 0.120 | 0.002570288 | 28.40 | 0.60 |
| Amygdala PGS (0.050) | Putamen (MRI) | 0.017 | -0.030 | 0.063 | 0.483080813 | 27.90 | 0.00 |
| Brainstem PGS (0.050) | Putamen (MRI) | 0.014 | -0.032 | 0.060 | 0.548972619 | 27.90 | 0.00 |
| Caudate PGS (0.050) | Putamen (MRI) | 0.062 | 0.016 | 0.107 | 0.007600293 | 28.30 | 0.40 |
| Pallidum PGS (0.050) | Putamen (MRI) | 0.121 | 0.074 | 0.167 | 3.5855E-07 | 29.40 | 1.60 |
| Putamen PGS (0.050) | Putamen (MRI) | 0.207 | 0.162 | 0.252 | 5.18228E-19 | 32.60 | 4.70 |
| Thalamus PGS (0.050) | Putamen (MRI) | 0.058 | 0.012 | 0.104 | 0.013895275 | 28.20 | 0.40 |
| Accumbens PGS (0.050) | Thalamus (MRI) | 0.037 | 0.000 | 0.075 | 0.050958372 | 56.00 | 0.10 |
| Amygdala PGS (0.050) | Thalamus (MRI) | 0.012 | -0.024 | 0.049 | 0.506107383 | 55.90 | 0.00 |
| Brainstem PGS (0.050) | Thalamus (MRI) | 0.054 | 0.018 | 0.090 | 0.003489599 | 56.20 | 0.30 |
| Caudate PGS (0.050) | Thalamus (MRI) | 0.035 | -0.001 | 0.071 | 0.058759694 | 56.00 | 0.10 |
| Pallidum PGS (0.050) | Thalamus (MRI) | 0.053 | 0.016 | 0.090 | 0.004846688 | 56.10 | 0.30 |
| Putamen PGS (0.050) | Thalamus (MRI) | 0.050 | 0.014 | 0.087 | 0.007196728 | 56.10 | 0.30 |
| Thalamus PGS (0.050) | Thalamus (MRI) | 0.155 | 0.119 | 0.191 | 4.35023E-17 | 58.40 | 2.60 |
| Accumbens PGS (0.100) | Accumbens (MRI) | 0.094 | 0.048 | 0.141 | 7.52914E-05 | 26.60 | 1.00 |
| Amygdala PGS (0.100) | Accumbens (MRI) | -0.022 | -0.068 | 0.023 | 0.333320223 | 25.70 | 0.10 |
| Brainstem PGS (0.100) | Accumbens (MRI) | 0.008 | -0.037 | 0.052 | 0.741677634 | 25.60 | 0.00 |
| Caudate PGS (0.100) | Accumbens (MRI) | 0.066 | 0.022 | 0.111 | 0.003670414 | 26.10 | 0.50 |
| Pallidum PGS (0.100) | Accumbens (MRI) | 0.056 | 0.011 | 0.102 | 0.015741627 | 26.00 | 0.40 |
| Putamen PGS (0.100) | Accumbens (MRI) | 0.077 | 0.032 | 0.123 | 0.000794182 | 26.30 | 0.70 |
| Thalamus PGS (0.100) | Accumbens (MRI) | 0.004 | -0.041 | 0.049 | 0.860141966 | 25.60 | 0.00 |
| Accumbens PGS (0.100) | Amygdala (MRI) | 0.030 | -0.014 | 0.074 | 0.179991846 | 41.00 | 0.10 |
| Amygdala PGS (0.100) | Amygdala (MRI) | 0.097 | 0.055 | 0.140 | 7.51679E-06 | 41.90 | 1.00 |
| Brainstem PGS (0.100) | Amygdala (MRI) | -0.004 | -0.046 | 0.039 | 0.86196905 | 40.90 | 0.00 |
| Caudate PGS (0.100) | Amygdala (MRI) | -0.013 | -0.055 | 0.029 | 0.549002771 | 40.90 | 0.00 |
| Pallidum PGS (0.100) | Amygdala (MRI) | 0.038 | -0.005 | 0.081 | 0.081710505 | 41.00 | 0.20 |
| Putamen PGS (0.100) | Amygdala (MRI) | 0.017 | -0.026 | 0.060 | 0.436658165 | 40.90 | 0.00 |
| Thalamus PGS (0.100) | Amygdala (MRI) | 0.005 | -0.038 | 0.047 | 0.829797952 | 40.90 | 0.00 |
| Accumbens PGS (0.100) | Brainstem (MRI) | 0.036 | -0.005 | 0.077 | 0.082887994 | 49.10 | 0.10 |
| Amygdala PGS (0.100) | Brainstem (MRI) | 0.013 | -0.026 | 0.053 | 0.511739512 | 49.00 | 0.00 |
| Brainstem PGS (0.100) | Brainstem (MRI) | 0.194 | 0.156 | 0.231 | 2.55566E-23 | 53.10 | 4.10 |
| Caudate PGS (0.100) | Brainstem (MRI) | 0.030 | -0.009 | 0.069 | 0.131878454 | 49.10 | 0.10 |
| Pallidum PGS (0.100) | Brainstem (MRI) | 0.084 | 0.045 | 0.124 | 3.16096E-05 | 49.80 | 0.70 |
| Putamen PGS (0.100) | Brainstem (MRI) | 0.053 | 0.013 | 0.092 | 0.008912408 | 49.30 | 0.30 |
| Thalamus PGS (0.100) | Brainstem (MRI) | 0.070 | 0.031 | 0.110 | 0.000436228 | 49.50 | 0.50 |
| Accumbens PGS (0.100) | Caudate (MRI) | 0.017 | -0.027 | 0.061 | 0.450829308 | 32.60 | 0.00 |
| Amygdala PGS (0.100) | Caudate (MRI) | 0.021 | -0.022 | 0.064 | 0.32788689 | 32.70 | 0.10 |
| Brainstem PGS (0.100) | Caudate (MRI) | -0.011 | -0.053 | 0.032 | 0.618884962 | 32.60 | 0.00 |
| Caudate PGS (0.100) | Caudate (MRI) | 0.147 | 0.105 | 0.188 | 7.01163E-12 | 35.30 | 2.60 |
| Pallidum PGS (0.100) | Caudate (MRI) | 0.058 | 0.015 | 0.101 | 0.008779817 | 33.00 | 0.40 |
| Putamen PGS (0.100) | Caudate (MRI) | 0.031 | -0.012 | 0.074 | 0.162062401 | 32.70 | 0.10 |
| Thalamus PGS (0.100) | Caudate (MRI) | -0.013 | -0.056 | 0.030 | 0.556640933 | 32.60 | 0.00 |
| Accumbens PGS (0.100) | Pallidum (MRI) | 0.060 | 0.014 | 0.105 | 0.00993564 | 33.70 | 0.40 |
| Amygdala PGS (0.100) | Pallidum (MRI) | 0.023 | -0.022 | 0.067 | 0.31665822 | 33.30 | 0.10 |
| Brainstem PGS (0.100) | Pallidum (MRI) | 0.064 | 0.021 | 0.108 | 0.00391549 | 33.70 | 0.50 |
| Caudate PGS (0.100) | Pallidum (MRI) | 0.067 | 0.023 | 0.110 | 0.002743859 | 33.80 | 0.50 |
| Pallidum PGS (0.100) | Pallidum (MRI) | 0.091 | 0.047 | 0.135 | 6.13844E-05 | 34.20 | 0.90 |
| Putamen PGS (0.100) | Pallidum (MRI) | 0.102 | 0.058 | 0.146 | 5.29818E-06 | 34.40 | 1.20 |
| Thalamus PGS (0.100) | Pallidum (MRI) | 0.033 | -0.011 | 0.077 | 0.1396035 | 33.40 | 0.10 |
| Accumbens PGS (0.100) | Putamen (MRI) | 0.071 | 0.024 | 0.119 | 0.003261962 | 28.40 | 0.50 |
| Amygdala PGS (0.100) | Putamen (MRI) | 0.017 | -0.029 | 0.064 | 0.461737273 | 27.90 | 0.00 |
| Brainstem PGS (0.100) | Putamen (MRI) | 0.017 | -0.029 | 0.062 | 0.475761848 | 27.90 | 0.00 |
| Caudate PGS (0.100) | Putamen (MRI) | 0.061 | 0.015 | 0.106 | 0.009058304 | 28.30 | 0.40 |
| Pallidum PGS (0.100) | Putamen (MRI) | 0.117 | 0.070 | 0.163 | 8.48601E-07 | 29.30 | 1.50 |
| Putamen PGS (0.100) | Putamen (MRI) | 0.204 | 0.159 | 0.249 | 1.34652E-18 | 32.40 | 4.60 |
| Thalamus PGS (0.100) | Putamen (MRI) | 0.050 | 0.004 | 0.096 | 0.032397132 | 28.10 | 0.30 |
| Accumbens PGS (0.100) | Thalamus (MRI) | 0.037 | -0.001 | 0.075 | 0.053295686 | 56.00 | 0.10 |
| Amygdala PGS (0.100) | Thalamus (MRI) | 0.011 | -0.026 | 0.048 | 0.561510679 | 55.90 | 0.00 |
| Brainstem PGS (0.100) | Thalamus (MRI) | 0.052 | 0.016 | 0.088 | 0.004771033 | 56.10 | 0.30 |
| Caudate PGS (0.100) | Thalamus (MRI) | 0.033 | -0.003 | 0.069 | 0.073429613 | 56.00 | 0.10 |
| Pallidum PGS (0.100) | Thalamus (MRI) | 0.054 | 0.017 | 0.091 | 0.004283774 | 56.10 | 0.30 |
| Putamen PGS (0.100) | Thalamus (MRI) | 0.053 | 0.017 | 0.090 | 0.004502109 | 56.10 | 0.30 |
| Thalamus PGS (0.100) | Thalamus (MRI) | 0.151 | 0.115 | 0.186 | 2.90625E-16 | 58.30 | 2.40 |
| Accumbens PGS (0.500) | Accumbens (MRI) | 0.090 | 0.043 | 0.137 | 0.000162746 | 26.50 | 0.90 |
| Amygdala PGS (0.500) | Accumbens (MRI) | -0.023 | -0.068 | 0.023 | 0.324230155 | 25.70 | 0.10 |
| Brainstem PGS (0.500) | Accumbens (MRI) | 0.010 | -0.034 | 0.055 | 0.649503606 | 25.60 | 0.00 |
| Caudate PGS (0.500) | Accumbens (MRI) | 0.061 | 0.016 | 0.106 | 0.007396771 | 26.00 | 0.50 |
| Pallidum PGS (0.500) | Accumbens (MRI) | 0.057 | 0.012 | 0.103 | 0.014148237 | 26.00 | 0.40 |
| Putamen PGS (0.500) | Accumbens (MRI) | 0.076 | 0.031 | 0.121 | 0.000988117 | 26.30 | 0.70 |
| Thalamus PGS (0.500) | Accumbens (MRI) | 0.001 | -0.044 | 0.047 | 0.948400275 | 25.60 | 0.00 |
| Accumbens PGS (0.500) | Amygdala (MRI) | 0.026 | -0.018 | 0.071 | 0.24533597 | 40.90 | 0.10 |
| Amygdala PGS (0.500) | Amygdala (MRI) | 0.096 | 0.053 | 0.138 | 1.04293E-05 | 41.80 | 1.00 |
| Brainstem PGS (0.500) | Amygdala (MRI) | -0.004 | -0.047 | 0.038 | 0.841755284 | 40.90 | 0.00 |
| Caudate PGS (0.500) | Amygdala (MRI) | -0.011 | -0.053 | 0.031 | 0.609531016 | 40.90 | 0.00 |
| Pallidum PGS (0.500) | Amygdala (MRI) | 0.038 | -0.006 | 0.081 | 0.087459611 | 41.00 | 0.10 |
| Putamen PGS (0.500) | Amygdala (MRI) | 0.015 | -0.028 | 0.058 | 0.492867984 | 40.90 | 0.00 |
| Thalamus PGS (0.500) | Amygdala (MRI) | 0.001 | -0.041 | 0.044 | 0.952118048 | 40.90 | 0.00 |
| Accumbens PGS (0.500) | Brainstem (MRI) | 0.036 | -0.005 | 0.076 | 0.087623452 | 49.10 | 0.10 |
| Amygdala PGS (0.500) | Brainstem (MRI) | 0.012 | -0.027 | 0.052 | 0.543122983 | 49.00 | 0.00 |
| Brainstem PGS (0.500) | Brainstem (MRI) | 0.183 | 0.145 | 0.220 | 8.03894E-21 | 52.70 | 3.70 |
| Caudate PGS (0.500) | Brainstem (MRI) | 0.031 | -0.008 | 0.070 | 0.115567042 | 49.10 | 0.10 |
| Pallidum PGS (0.500) | Brainstem (MRI) | 0.085 | 0.046 | 0.125 | 2.42056E-05 | 49.80 | 0.80 |
| Putamen PGS (0.500) | Brainstem (MRI) | 0.057 | 0.018 | 0.096 | 0.004578934 | 49.40 | 0.30 |
| Thalamus PGS (0.500) | Brainstem (MRI) | 0.067 | 0.028 | 0.106 | 0.000805365 | 49.50 | 0.50 |
| Accumbens PGS (0.500) | Caudate (MRI) | 0.016 | -0.029 | 0.060 | 0.49403084 | 32.60 | 0.00 |
| Amygdala PGS (0.500) | Caudate (MRI) | 0.021 | -0.022 | 0.064 | 0.343916048 | 32.70 | 0.10 |
| Brainstem PGS (0.500) | Caudate (MRI) | -0.006 | -0.049 | 0.036 | 0.771826776 | 32.60 | 0.00 |
| Caudate PGS (0.500) | Caudate (MRI) | 0.136 | 0.094 | 0.178 | 2.44834E-10 | 34.90 | 2.30 |
| Pallidum PGS (0.500) | Caudate (MRI) | 0.057 | 0.014 | 0.100 | 0.009932516 | 33.00 | 0.40 |
| Putamen PGS (0.500) | Caudate (MRI) | 0.028 | -0.015 | 0.071 | 0.199611178 | 32.70 | 0.10 |
| Thalamus PGS (0.500) | Caudate (MRI) | -0.015 | -0.058 | 0.028 | 0.485087238 | 32.60 | 0.00 |
| Accumbens PGS (0.500) | Pallidum (MRI) | 0.058 | 0.013 | 0.104 | 0.012428884 | 33.60 | 0.40 |
| Amygdala PGS (0.500) | Pallidum (MRI) | 0.022 | -0.022 | 0.066 | 0.323115666 | 33.30 | 0.10 |
| Brainstem PGS (0.500) | Pallidum (MRI) | 0.063 | 0.020 | 0.107 | 0.004535571 | 33.70 | 0.50 |
| Caudate PGS (0.500) | Pallidum (MRI) | 0.063 | 0.020 | 0.107 | 0.004303468 | 33.70 | 0.50 |
| Pallidum PGS (0.500) | Pallidum (MRI) | 0.089 | 0.044 | 0.133 | 9.26168E-05 | 34.10 | 0.90 |
| Putamen PGS (0.500) | Pallidum (MRI) | 0.101 | 0.057 | 0.144 | 7.10572E-06 | 34.40 | 1.10 |
| Thalamus PGS (0.500) | Pallidum (MRI) | 0.030 | -0.013 | 0.074 | 0.173711071 | 33.40 | 0.10 |
| Accumbens PGS (0.500) | Putamen (MRI) | 0.069 | 0.021 | 0.117 | 0.0045854 | 28.30 | 0.50 |
| Amygdala PGS (0.500) | Putamen (MRI) | 0.018 | -0.028 | 0.065 | 0.437725789 | 27.90 | 0.00 |
| Brainstem PGS (0.500) | Putamen (MRI) | 0.021 | -0.025 | 0.066 | 0.373593808 | 27.90 | 0.00 |
| Caudate PGS (0.500) | Putamen (MRI) | 0.058 | 0.013 | 0.104 | 0.012273656 | 28.20 | 0.40 |
| Pallidum PGS (0.500) | Putamen (MRI) | 0.113 | 0.067 | 0.160 | 1.71195E-06 | 29.20 | 1.40 |
| Putamen PGS (0.500) | Putamen (MRI) | 0.192 | 0.147 | 0.237 | 1.25728E-16 | 31.90 | 4.10 |
| Thalamus PGS (0.500) | Putamen (MRI) | 0.045 | -0.001 | 0.091 | 0.055528218 | 28.10 | 0.20 |
| Accumbens PGS (0.500) | Thalamus (MRI) | 0.037 | -0.001 | 0.075 | 0.058947443 | 56.00 | 0.10 |
| Amygdala PGS (0.500) | Thalamus (MRI) | 0.010 | -0.027 | 0.047 | 0.599090557 | 55.90 | 0.00 |
| Brainstem PGS (0.500) | Thalamus (MRI) | 0.052 | 0.015 | 0.088 | 0.005188203 | 56.10 | 0.30 |
| Caudate PGS (0.500) | Thalamus (MRI) | 0.029 | -0.008 | 0.065 | 0.120852302 | 55.90 | 0.10 |
| Pallidum PGS (0.500) | Thalamus (MRI) | 0.054 | 0.017 | 0.091 | 0.004225999 | 56.10 | 0.30 |
| Putamen PGS (0.500) | Thalamus (MRI) | 0.057 | 0.020 | 0.094 | 0.002347627 | 56.20 | 0.30 |
| Thalamus PGS (0.500) | Thalamus (MRI) | 0.145 | 0.110 | 0.181 | 3.09809E-15 | 58.10 | 2.30 |
| Accumbens PGS (1.000) | Accumbens (MRI) | 0.089 | 0.043 | 0.136 | 0.000181541 | 26.50 | 0.90 |
| Amygdala PGS (1.000) | Accumbens (MRI) | -0.023 | -0.068 | 0.023 | 0.323338648 | 25.70 | 0.10 |
| Brainstem PGS (1.000) | Accumbens (MRI) | 0.011 | -0.034 | 0.055 | 0.643295772 | 25.60 | 0.00 |
| Caudate PGS (1.000) | Accumbens (MRI) | 0.060 | 0.016 | 0.105 | 0.008044272 | 26.00 | 0.40 |
| Pallidum PGS (1.000) | Accumbens (MRI) | 0.057 | 0.012 | 0.103 | 0.01417541 | 26.00 | 0.40 |
| Putamen PGS (1.000) | Accumbens (MRI) | 0.075 | 0.030 | 0.120 | 0.001155981 | 26.30 | 0.70 |
| Thalamus PGS (1.000) | Accumbens (MRI) | 0.001 | -0.044 | 0.046 | 0.96017824 | 25.60 | 0.00 |
| Accumbens PGS (1.000) | Amygdala (MRI) | 0.026 | -0.019 | 0.070 | 0.25560944 | 40.90 | 0.10 |
| Amygdala PGS (1.000) | Amygdala (MRI) | 0.095 | 0.053 | 0.138 | 1.12134E-05 | 41.80 | 1.00 |
| Brainstem PGS (1.000) | Amygdala (MRI) | -0.004 | -0.046 | 0.038 | 0.850050279 | 40.90 | 0.00 |
| Caudate PGS (1.000) | Amygdala (MRI) | -0.011 | -0.053 | 0.032 | 0.622166633 | 40.90 | 0.00 |
| Pallidum PGS (1.000) | Amygdala (MRI) | 0.038 | -0.006 | 0.081 | 0.088318521 | 41.00 | 0.10 |
| Putamen PGS (1.000) | Amygdala (MRI) | 0.014 | -0.029 | 0.057 | 0.51921607 | 40.90 | 0.00 |
| Thalamus PGS (1.000) | Amygdala (MRI) | 0.001 | -0.042 | 0.043 | 0.967430114 | 40.90 | 0.00 |
| Accumbens PGS (1.000) | Brainstem (MRI) | 0.035 | -0.005 | 0.076 | 0.089332161 | 49.10 | 0.10 |
| Amygdala PGS (1.000) | Brainstem (MRI) | 0.012 | -0.027 | 0.052 | 0.546526202 | 49.00 | 0.00 |
| Brainstem PGS (1.000) | Brainstem (MRI) | 0.181 | 0.143 | 0.219 | 2.17882E-20 | 52.60 | 3.60 |
| Caudate PGS (1.000) | Brainstem (MRI) | 0.032 | -0.007 | 0.071 | 0.112699413 | 49.10 | 0.10 |
| Pallidum PGS (1.000) | Brainstem (MRI) | 0.086 | 0.046 | 0.125 | 2.35639E-05 | 49.80 | 0.80 |
| Putamen PGS (1.000) | Brainstem (MRI) | 0.057 | 0.018 | 0.097 | 0.004342092 | 49.40 | 0.40 |
| Thalamus PGS (1.000) | Brainstem (MRI) | 0.067 | 0.027 | 0.106 | 0.00086759 | 49.50 | 0.50 |
| Accumbens PGS (1.000) | Caudate (MRI) | 0.015 | -0.029 | 0.060 | 0.499737114 | 32.60 | 0.00 |
| Amygdala PGS (1.000) | Caudate (MRI) | 0.020 | -0.022 | 0.063 | 0.350003944 | 32.70 | 0.10 |
| Brainstem PGS (1.000) | Caudate (MRI) | -0.006 | -0.048 | 0.037 | 0.791744026 | 32.60 | 0.00 |
| Caudate PGS (1.000) | Caudate (MRI) | 0.134 | 0.093 | 0.176 | 3.82812E-10 | 34.80 | 2.20 |
| Pallidum PGS (1.000) | Caudate (MRI) | 0.057 | 0.014 | 0.100 | 0.009946856 | 33.00 | 0.40 |
| Putamen PGS (1.000) | Caudate (MRI) | 0.027 | -0.016 | 0.070 | 0.217916171 | 32.70 | 0.10 |
| Thalamus PGS (1.000) | Caudate (MRI) | -0.016 | -0.058 | 0.027 | 0.471843708 | 32.60 | 0.00 |
| Accumbens PGS (1.000) | Pallidum (MRI) | 0.058 | 0.012 | 0.104 | 0.012966943 | 33.60 | 0.30 |
| Amygdala PGS (1.000) | Pallidum (MRI) | 0.022 | -0.022 | 0.066 | 0.32499406 | 33.30 | 0.10 |
| Brainstem PGS (1.000) | Pallidum (MRI) | 0.063 | 0.019 | 0.106 | 0.004732019 | 33.70 | 0.50 |
| Caudate PGS (1.000) | Pallidum (MRI) | 0.063 | 0.020 | 0.107 | 0.004471602 | 33.70 | 0.50 |
| Pallidum PGS (1.000) | Pallidum (MRI) | 0.088 | 0.044 | 0.133 | 9.58383E-05 | 34.10 | 0.90 |
| Putamen PGS (1.000) | Pallidum (MRI) | 0.100 | 0.056 | 0.143 | 9.05108E-06 | 34.40 | 1.10 |
| Thalamus PGS (1.000) | Pallidum (MRI) | 0.030 | -0.014 | 0.074 | 0.182159603 | 33.40 | 0.10 |
| Accumbens PGS (1.000) | Putamen (MRI) | 0.069 | 0.021 | 0.116 | 0.004894161 | 28.30 | 0.50 |
| Amygdala PGS (1.000) | Putamen (MRI) | 0.018 | -0.028 | 0.065 | 0.436793368 | 27.90 | 0.00 |
| Brainstem PGS (1.000) | Putamen (MRI) | 0.021 | -0.024 | 0.067 | 0.360599857 | 27.90 | 0.10 |
| Caudate PGS (1.000) | Putamen (MRI) | 0.058 | 0.013 | 0.104 | 0.012402736 | 28.20 | 0.40 |
| Pallidum PGS (1.000) | Putamen (MRI) | 0.113 | 0.067 | 0.159 | 1.83101E-06 | 29.20 | 1.40 |
| Putamen PGS (1.000) | Putamen (MRI) | 0.189 | 0.144 | 0.234 | 3.76615E-16 | 31.80 | 4.00 |
| Thalamus PGS (1.000) | Putamen (MRI) | 0.044 | -0.002 | 0.090 | 0.060211185 | 28.10 | 0.20 |
| Accumbens PGS (1.000) | Thalamus (MRI) | 0.037 | -0.001 | 0.075 | 0.059534092 | 56.00 | 0.10 |
| Amygdala PGS (1.000) | Thalamus (MRI) | 0.010 | -0.027 | 0.047 | 0.603584813 | 55.90 | 0.00 |
| Brainstem PGS (1.000) | Thalamus (MRI) | 0.052 | 0.015 | 0.088 | 0.005222413 | 56.10 | 0.30 |
| Caudate PGS (1.000) | Thalamus (MRI) | 0.028 | -0.008 | 0.065 | 0.127951066 | 55.90 | 0.10 |
| Pallidum PGS (1.000) | Thalamus (MRI) | 0.054 | 0.017 | 0.091 | 0.004223716 | 56.10 | 0.30 |
| Putamen PGS (1.000) | Thalamus (MRI) | 0.057 | 0.021 | 0.094 | 0.002161912 | 56.20 | 0.40 |
| Thalamus PGS (1.000) | Thalamus (MRI) | 0.145 | 0.109 | 0.180 | 3.96067E-15 | 58.10 | 2.30 |
