# Supplementary material for: Genetic scores for adult subcortical volumes associate with subcortical volumes during infancy and childhood
Source: Hum Brain Mapp. 2021 Feb 2;42(6):1583–93. doi: 10.1002/hbm.25292 (PMC7978120; doi:10.1002/hbm.25292)
Supplement: Supplementary file 9 — Table S2 Full results for the associations of the GTO PGS with the GTOD from ultrasound and the MRI‐based GTO‐like volume. [file HBM-42-1583-s005.docx]

**Supplementary Table 2. Full results for the associations of the GTO PGS with the GTOD from ultrasound and the MRI-based GTO-like volume.**

| **Determinant** | **Outcome** | **Beta** | **95% CI (lower)** | **95% CI (upper)** | **p-value** | **R2 baseline (%)** | **R2 change (%)** |
| --- | --- | --- | --- | --- | --- | --- | --- |
| GTO PGS (0.005) | GTOD (US) | 0.102 | 0.033 | 0.171 | 0.003926949 | 30.50 | 1.10 |
| GTO PGS (0.010) | GTOD (US) | 0.109 | 0.041 | 0.177 | 0.001771545 | 30.70 | 1.30 |
| GTO PGS (0.050) | GTOD (US) | 0.112 | 0.045 | 0.179 | 0.001059572 | 30.80 | 1.40 |
| GTO PGS (0.100) | GTOD (US) | 0.110 | 0.043 | 0.176 | 0.001271025 | 30.70 | 1.40 |
| GTO PGS (0.500) | GTOD (US) | 0.106 | 0.039 | 0.172 | 0.001907048 | 30.60 | 1.30 |
| GTO PGS (1.000) | GTOD (US) | 0.105 | 0.039 | 0.171 | 0.002001498 | 30.60 | 1.30 |
| GTO PGS (0.005) | GTO-like volume (MRI) | 0.145 | 0.112 | 0.178 | 2.8431E-17 | 61.40 | 2.40 |
| GTO PGS (0.010) | GTO-like volume (MRI) | 0.147 | 0.114 | 0.180 | 9.61711E-18 | 61.40 | 2.50 |
| GTO PGS (0.050) | GTO-like volume (MRI) | 0.137 | 0.104 | 0.170 | 1.06927E-15 | 61.10 | 2.20 |
| GTO PGS (0.100) | GTO-like volume (MRI) | 0.132 | 0.099 | 0.165 | 1.06568E-14 | 61.00 | 2.00 |
| GTO PGS (0.500) | GTO-like volume (MRI) | 0.125 | 0.092 | 0.158 | 2.19108E-13 | 60.80 | 1.80 |
| GTO PGS (1.000) | GTO-like volume (MRI) | 0.124 | 0.091 | 0.157 | 3.64803E-13 | 60.70 | 1.80 |
