# Supplementary material for: Genetic scores for adult subcortical volumes associate with subcortical volumes during infancy and childhood
Source: Hum Brain Mapp. 2021 Feb 2;42(6):1583–93. doi: 10.1002/hbm.25292 (PMC7978120; doi:10.1002/hbm.25292)
Supplement: Supplementary file 10 — Table S3 Mediation results for the mediation models where the GTO‐like volume is replaced with an estimated diameter based on a spherical model. [file HBM-42-1583-s001.docx]

**Supplementary Table 3. Mediation results for the mediation models where the GTO-like volume is replaced with an estimated diameter based on a spherical model.**

| **Parameter P** | **Direct effect (95% CI)** | **Indirect effect (95% CI)** | **p-value** |
| --- | --- | --- | --- |
| 0.005 | 87.1% (67.5 – 100.0) | 12.9% (0.0 – 32.5) | 0.160 |
| 0.010 | 85.6% (66.6 – 100.0) | 14.4% (0.0 – 33.4) | 0.070 |
| 0.050 | 83.2% (61.2 – 97.4) | 16.8% (2.6 – 38.8) | 0.018 |
| 0.100 | 82.8% (58.6 – 97.9) | 17.2% (2.1 – 41.4) | 0.026 |
| 0.500 | 82.3% (56.2 – 97.6) | 17.7% (2.4 – 43.8) | 0.020 |
| 1.000 | 82.2% (56.2 – 97.5) | 17.8% (2.5 – 43.8) | 0.032 |
